# Third Exposure to a Reduced Carbohydrate Meal Lowers Evening Postprandial Insulin and GIP Responses and HOMA-IR Estimate of Insulin Resistance

**DOI:** 10.1371/journal.pone.0165378

**Published:** 2016-10-31

**Authors:** Po-Ju Lin, Katarina T. Borer

**Affiliations:** School of Kinesiology, University of Michigan, Ann Arbor, Michigan, United States of America; University of Bath, UNITED KINGDOM

## Abstract

**Background:**

Postprandial hyperinsulinemia, hyperglycemia, and insulin resistance increase the risk of type 2 diabetes (T2D) and cardiovascular disease mortality. Postprandial hyperinsulinemia and hyperglycemia also occur in metabolically healthy subjects consuming high-carbohydrate diets particularly after evening meals and when carbohydrate loads follow acute exercise. We hypothesized the involvement of dietary carbohydrate load, especially when timed after exercise, and mediation by the glucose-dependent insulinotropic peptide (GIP) in this phenomenon, as this incretin promotes insulin secretion after carbohydrate intake in insulin-sensitive, but not in insulin-resistant states.

**Methods:**

Four groups of eight metabolically healthy weight-matched postmenopausal women were provided with three isocaloric meals (a pre-trial meal and two meals during the trial day) containing either 30% or 60% carbohydrate, with and without two-hours of moderate-intensity exercise before the last two meals. Plasma glucose, insulin, glucagon, GIP, glucagon-like peptide 1 (GLP-1), free fatty acids (FFAs), and D-3-hydroxybutyrate concentrations were measured during 4-h postprandial periods and 3-h exercise periods, and their areas under the curve (AUCs) were analyzed by mixed-model ANOVA, and insulin resistance during fasting and meal tolerance tests within each diet was estimated using homeostasis-model assessment (HOMA-IR).

**Results:**

The third low-carbohydrate meal, but not the high-carbohydrate meal, reduced: (1) evening insulin AUC by 39% without exercise and by 31% after exercise; (2) GIP AUC by 48% without exercise and by 45% after exercise, and (3) evening insulin resistance by 37% without exercise and by 24% after exercise. Pre-meal exercise did not alter insulin-, GIP- and HOMA-IR- lowering effects of low-carbohydrate diet, but exacerbated evening hyperglycemia.

**Conclusions:**

Evening postprandial insulin and GIP responses and insulin resistance declined by over 30% after three meals that limited daily carbohydrate intake to 30% compared to no such changes after three 60%-carbohydrate meals, an effect that was independent of pre-meal exercise. The parallel timing and magnitude of postprandial insulin and GIP changes suggest their dependence on a delayed intestinal adaptation to a low-carbohydrate diet. Pre-meal exercise exacerbated glucose intolerance with both diets most likely due to impairment of insulin signaling by pre-meal elevation of FFAs.

## Introduction

Postprandial hyperglycemia (>7.8 mmol/L) is a diagnostic symptom of type 2 diabetes (T2D) where it is prevalent throughout the day [1] and is accompanied with hyperinsulinemia. Both the persistent hyperglycemia as reflected in elevated hemoglobin A1c [2,3] and hyperinsulinemia [2–4] have been associated with adverse coronary heart disease (CHD) risk profile [4] and, in prospective epidemiological studies, with increased risk of CHD [5,6] and increased mortality [2,3]. The association of hyperglycemia and hyperinsulinemia in T2D with obesity is firmly established as both can be reduced with weight loss [7,8]. However, it is unclear whether the rise in T2D incidence from 4.4 million or 2.4% of the US population in 1970s [9] to 29.1 million or 9.3% of the population in 2014 [10] may have been facilitated by a 30.5% increase in daily carbohydrate consumption from 213 g per day in 1965 to 278 g per day or 51% of daily calories in 2011 [11]. The currently high carbohydrate consumption falls within the 45 to 65% of daily calorie range recommended in 2010 by Departments of Agriculture and Health and Human Services [12]. This study explores the extent to which the recommended high daily carbohydrate intake contributes to evening postprandial glucose intolerance. Postprandial hyperglycemia and delayed or protracted hyperinsulinemia are prevalent in metabolically healthy individuals in the evening but not in the morning when the same carbohydrate load is delivered as oral glucose [13–15], a carbohydrate-containing meal [16], or as intravenous glucose injection [17]. Insulin hypoglycemic action for the same carbohydrate load also is lower in the evening than in the morning as shown by the requirement for a higher evening dose of insulin secretagogue tolbutamide [13] and of insulin in type 1 diabetics [18]. A circadian influence was recently implicated in the reduced β cell capacity to secrete insulin in the evening [19]. However, neither this study nor two others [20,21] eliminated the possibility that the evening postprandial hyperglycemia and delayed or protracted hyperinsulinemia result from the large daily cumulative carbohydrate load.

Postprandial hyperglycemia and hyperinsulinemia also may be affected by the particular timing of exercise with respect to high-carbohydrate meals. It is established that acute exercise increases glucose tolerance by facilitating muscle glucose uptake through a non-insulin-dependent mechanism in both metabolically healthy [22] and T2D subjects [23]. Acute exercise also increases muscle sensitivity to insulin for a number of hours shortly after a bout of exercise [24,25]. However, acute exercise performed before an oral glucose tolerance test or a high-carbohydrate meal can lead to glucose intolerance [26,27]. This glucose intolerance is the result of the compounding contribution of absorbed ingested glucose to residual exercise-induced endogenous glucose production by the liver [26,27] and is exacerbated at higher exercise intensities by increased secretion of catecholamines [28]. We have, therefore, included a bout of moderate-intensity exercise before the two trial meals to assess its possible contribution to postprandial glucose intolerance and compensatory insulin response after meals that differ in carbohydrate content. Postprandial concentrations of incretin gut peptides, glucose-dependent insulinotropic peptide (GIP) and glucagon-like peptide 1 (GLP-1), were measured as these two incretins amplify postprandial insulin secretion and suppress glucagon secretion [29] when glucose is delivered by oral rather than by intravenous route [30]. GIP and GLP-1 are released, respectively, from the proximal and distal small intestine in response to ingested carbohydrates [31] and fat [32]. Their importance in the control of insulin secretion is underscored not only by their insulinotropic properties in insulin-sensitive individuals but also by the impairment of their action in experimentally-induced insulin resistance [33] and T2D [34–37].

The principal aim of this study was to examine the contribution of the dietary carbohydrate to the evening postprandial hyperglycemia and compensatory insulin secretion by comparing postprandial effects of carbohydrate intake at the currently recommended levels [12], to a lower daily load that was found to be beneficial in lowering glycemia and hemoglobin A1c in individuals with T2D [38]. The secondary aim was to assess whether two hours of pre-meal moderate-intensity exercise would affect any diet-induced glucose- and insulin-lowering effect. And the final aim was to determine whether any diet-induced changes in postprandial insulin secretion and glycemia may be related to changes in the secretion of incretin hormones. Our three hypotheses were that (1) reducing the carbohydrate content of the diet from 60% to 30% will reduce postprandial glycemia and insulin responses at both meals and abolish evening postprandial hyperglycemia; (2) exercise will have an independent effect on postprandial glucose tolerance and insulin response; and (3) changes in postprandial insulin responses will be affected by incretins GIP and GLP-1. We chose a repeat-event, rather than the customary single-event, experimental design to better approximate human habitual intermittent diurnal feeding and locomotor patterns.

## Methods

### Subjects

Subject were 32 healthy postmenopausal women, 50 to 65 years old; of body mass index (BMI) 20 to 30 kg/m^2^; with a fasting glucose level < 100 mg/dL; hematocrit > 32%, hemoglobin > 12 mg/dL; non-smokers; and without endocrine and metabolic disorders. They were volunteers recruited from the University of Michigan clinical studies webpage (UMClinicalStudies.org). All subjects signed an informed consent for human clinical studies HUM00017875 and HUM00043233 approved by The University of Michigan Medical School Institutional Review Board (IRB-MED) on 8/7/2008 and 11/11/2010, respectively. These constituent studies were registered as a clinical trial NCT01891617 with ClinicalTrials.gov after the initiation of the subject recruitment in November 2008. All subject recruitment was completed by August of 2012. The authors confirm that all trials for this intervention were registered.

### General experimental protocol

Subjects underwent preliminary health and fitness screens at the Michigan Clinical Research Unit (MCRU). The health screen included health history, measurements of weight, height, and body fat by a dual-energy X-ray absorptiometry (General Electric Lunar Prodigy Advance), and a fasting blood draw for fasting glucose and other laboratory chemistries. A fitness screen assessed individual maximal aerobic effort. It consisted of a treadmill test at 3 miles per hour with 2% slope increments every 3 minutes with the subject breathing through a mouthpiece using a Max II metabolic cart (AEI Technologies, Inc., Bastrop, TX). The criterion of maximal effort used was a respiratory quotient of 1. After matching by body weight, BMI, and aerobic fitness, 16 subjects, each, were assigned to low-carbohydrate (LC) or high-carbohydrate (HC) meals, and, within each dietary group, 8 subjects participated either in a sedentary (LCS or HCS) or an exercise (LCX or HCX) trial.

### Study protocol (Fig 1)

**Fig 1:**
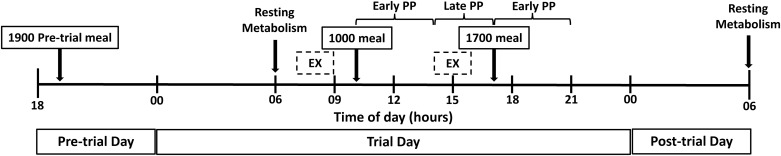
Study protocol. EX, 2-hour moderate-intensity exercise in the exercise trial; Early PP, early postprandial period; Late PP, late postprandial period.

At 1800 h, the evening before the study day, subjects were admitted to MCRU. A meal containing 10 kcal/kg body weight was provided at 1900 h with the macronutrient composition appropriate to the assigned dietary group. Hourly blood collection over 24 hours was initiated at 0600 h on the study day through an antecubital vein catheter kept patent with sodium heparin. Additional samples were taken at 15- and 30-min intervals during meals and exercise. Plasma glucose, insulin, glucagon, GIP and GLP-1 responses were examined as a function of meal carbohydrate content in the presence or absence of exercise. The free fatty acids (FFAs) and D-3-hydroxybutyrate concentrations also were measured as they are elicited by reduced glucose availability after LC meals and can impair insulin sensitivity [39].

### Meals

In addition to the pre-trial evening meal, two isocaloric meals were provided at 1000 and 1700 h on the trial day (Fig 1), each containing 12.5 kcal/kg body-weight. Within each dietary group, the same dietary composition was provided. In the LC meals (30% carbohydrate, 25% protein, and 45% fat), the food items provided in the morning were macaroni and cheese, sausage and ham, wheat bread roll with butter, Chef’s salad (romaine lettuce, tomatoes, cucumbers, slivered turkey and ham strips, cheddar and Swiss cheese, croutons, French salad dressing), fruit-flavored yogurt with shredded almonds, and fruit. The glycemic index (GI) of the morning LC meal, calculated with the Nutrition Data System for Research (NDSR) was 53. The food items provided in the LC meal in the evening were a veggie burger, minestrone soup, Caesar salad with chicken and almonds, fruit juice and fruit. The GI of the evening LC meal was 51. Sugar content of dietary carbohydrates in the three LC meals identified from NDSR was about 28 g or 54% per meal, and the type of fat was between about 10 and 14 g, each, of saturated, monounsaturated and polyunsaturated fatty acids. The HC meals (60% carbohydrate, 15% protein, and 25% fat) included in the morning an egg salad on multigrain bun, whole-wheat rolls with butter, coleslaw, carrot sticks, bananas, orange juice, skim milk, and graham crackers. The GI of the morning HC meal was 58. The food items provided in the HC meal in the evening were a bacon, ham, and cheese sandwich (wheat toast, slivered ham, bacon, cheddar cheese, tomatoes, lettuce, diet mayonnaise), mixed cooked vegetables (broccoli, cauliflower, and carrots), tossed green salad, fruit juice, pretzels, and vanilla ice cream. The GI of the evening HC meal was 68. Sugar content of dietary carbohydrates in the three HC meals was about 49 g or 43% per meal, and the type of fat was between about 6 and 7 g, each, of saturated, monounsaturated and polyunsaturated fatty acids. Food provided and any left uneaten was weighed to determine energy and nutrient consumption.

### Exercise

Two-hour treadmill walking took place from 0700 to 0900 h and from 1400 to 1600 h at 45% VO_2_ max and was completed 1 hour before each meal (Fig 1). Walking speed was 3 miles/hour with modifications of the treadmill incline to adjust exercise intensity.

### Indirect calorimetry

Resting metabolism was measured by indirect calorimetry between 0600–0630 h on the trial and post-trial days (Fig 1) with a head canopy (Viasys apparatus, Respiratory Care Inc., Yorba Linda, CA). Exercise metabolism was measured by indirect calorimetry during the first 30 minutes of each exercise hour. Energy expenditure and substrate utilization during rest and exercise were estimated using the Weir equation [40].

### Analytical procedures

Blood samples were collected into ice-chilled EDTA-coated tubes containing aprotinin (50 KIU/mL blood, Sigma Chemical, St. Louis, MO) and dipeptidyl peptidase-4 inhibitor (10 μL/mL blood; EMD Millipore Corporation, Billerica, MA). Plasma was kept frozen at -80°C for hormone and metabolite measurements. Plasma glucose (Fisher Diagnostics, Middletown, VA) and FFAs (Wako Diagnostics, Richmond, VA) were measured with enzymatic colorimetric assays, and D-3-hydroxybutyrate with a kinetic enzymatic method (Randox Laboratories-US, Ltd., Kearneysville, WV). Plasma insulin and glucagon were measured with radioimmunoassays (EMD Millipore Corporation, Billerica, MA), and GIP and GLP-1 with a milliplex chemiluminescent assay kit (HGT-68K, EMD Millipore Corporation, Billerica, MA). The intra-assay coefficients of variation (CV) for insulin and glucagon were respectively 2.3% and 3.6% and inter-assay CVs were 16.2% for both. For GIP and GLP-1, intra- and inter-assay CVs were <11% and <19%, respectively.

### Statistical analyses

Data are presented as the mean and the standard error. Hormone and metabolite areas under the curves (AUCs) were calculated by the trapezoid rule during the 4-hour early PPs (1000–1400 h and 1700–2100 h) and the 3-hour late PP (1400–1700 h) in the sedentary trial, and during exercise periods (0700–1000 h and 1400–1700 h) that included 2 hours of exercise and 1 post-exercise hour. Subject characteristics, energy consumption and expenditure, and all measured hormone and metabolites AUCs were tested for normality with Shapiro-Wilk test (W statistic), and the normally distributed data were subjected to analysis of variance (ANOVA, F statistic) using Statistical Analysis System program (SAS version 9.3, SAS Institute, Cary, NC). Mixed-model repeated-measures ANOVA was used to analyze the effects of diurnal meal timing and diet composition. Postprandial AUCs between LC and HC groups were analyzed as between-subject effects, while the diurnal meal timing (morning vs. evening) and the interaction between the meal timing and diet composition were analyzed as within-subject effects. For group variables that were not normally distributed, Wilcoxon rank-sum (Z statistic) test was used, and their time effects were tested with Wilcoxon signed-rank test (S statistic). Insulin resistance during the postprandial periods within each dietary condition in the presence and the absence of exercise was estimated with the homeostatic model assessment (HOMA-IR) [41] test for the meal tolerance validated against the minimal model [42] and the intravenous glucose tolerance test [43].

## Results

### Energy intake and metabolism

The four experimental groups did not differ with respect to age, weight, percent body fat, BMI, or the level of aerobic fitness (Tables 1 and 2). The pre-trial fasting glucose concentration was assessed during the preliminary health-screen visit and was not different in the 4 experimental groups (LCS: 88.8 ± 3.0, HCS: 96.2 ± 4.1, LCX: 90.7 ± 1.6, HCX: 94.7 ± 2.2). Both the mean energy intake (783.4 ± 20.9 Kcal per meal) and energy expended during exercise (445.0 ± 22.5 Kcal per bout) in either trial were unaffected by diet composition, time of day, or exercise (Tables 1 and 2). However, the pre-trial meal composition affected fuel utilization in the two exercise trials (Table 2). Carbohydrate utilization was higher in HCX than in LCX during the 0600 fast (36% higher, F = 4.69, p = 0.05) and during afternoon exercise (61% vs 42% of energy, F = 11.65, p<0.01). In addition, HCX subjects utilized more carbohydrate during the afternoon than the morning exercise (61% vs 43% of energy, F = 27.03, p<0.01). During the post-trial morning fast at 0600 h, HCX subjects utilized 25% less carbohydrate and 38% more fat than in the morning of the trial day (F = 6.21, p = 0.03).

**Table 1:** Subject characteristics and energy intake in sedentary trials with low-carbohydrate (LCS) and high-carbohydrate meals (HCS).

Groups	LCS (n = 8)	HCS (n = 8)
**Age (years)**	**56.9 ± 1.54**	**55.0 ± 1.07**
**Weight (Kg)**	**69.9 ± 3.41**	**66.1 ± 2.22**
**Percentage of body fat (%)**	**38.0 ± 1.65**	**35.1 ± 2.18**
**BMI (Kg/m**^**2**^**)**	**25.4 ± 0.75**	**23.6 ± 0.91**
**Fitness level (VO**_**2**_**/min×Kg)**	**24.7 ± 2.49**	**25.6 ± 3.66**
**Trial-day fasting glucose (mg/dL)**	**82.7 ± 4.23**	**78.0 ± 2.43**
**Trial-day fasting insulin (microU/mL)**	**14.9 ± 1.39**	**10.6 ± 1.47**
**Trial-day resting carbohydrate utilization (%)**	**41.7 ± 14.95**	**48.3 ± 21.65**
**Trial-day resting fat utilization (%)**	**58.3 ± 14.95**	**51.7 ± 21.65**
**EI in meal 1 (Kcal)**	**751.8 ± 59.69**	**769.5 ± 32.82**
**EI in meal 2 (Kcal)**	**648.6 ± 112.98**	**803.8 ± 33.69**
**Post-trial resting carbohydrate utilization (%)**	**37.3 ± 8.73**	**50.5 ± 22.72**
**Post-trial resting fat utilization (%)**	**62.7 ± 8.73**	**49.5 ± 22.72**

LCS, sedentary trial with low-carbohydrate meals; HCS, sedentary trial with high-carbohydrate meals; BMI, body mass index; EI, energy intake.

**Table 2:** Subject characteristics and energy balance in exercise trials with low-carbohydrate (LCX) and high-carbohydrate meals (HCX).

Groups	LCX (n = 8)	HCX (n = 8)
**Age (years)**	**59.3 ± 1.46**	**59.9 ± 1.03**
**Weight (Kg)**	**71.8 ± 2.76**	**65.9 ± 3.19**
**Percentage of body fat (%)**	**39.0 ± 3.30**	**36.9 ± 2.65**
**BMI (Kg/m**^**2**^**)**	**25.7 ± 1.16**	**24.1 ± 0.90**
**Fitness level (VO**_**2**_**/min×Kg)**	**26.2 ± 3.60**	**22.6 ± 1.79**
**Trial-day fasting glucose (mg/dL)**	**82.4 ± 4.81**	**78.7 ± 3.57**
**Trial-day fasting insulin (microU/mL)**	**16.6 ± 1.56**^a^	**9.5 ± 0.46**^b^
**Trial-day resting carbohydrate utilization (%)**	**44.0 ± 12.04**^a^	**60.1 ± 18.03**^b^
**Trial-day resting fat utilization (%)**	**55.9 ± 12.09**^a^	**39.9 ± 18.03**^b^
**EI in meal 1 (Kcal)**	**782.1 ± 58.24**	**816.4 ± 43.14**
**EI in meal 2 (Kcal)**	**873.2 ± 38.00**	**821.7 ± 41.72**
**1**^**st**^ **Exercise EE (Kcal)**	**485.2 ± 63.28**	**413.7 ± 24.55**
**Carbohydrate utilization (%) during 1**^**st**^ **exercise**	**40.8 ± 3.84**	**42.5 ± 5.44**
**Fat utilization (%) during 1**^**st**^ **exercise**	**59.2 ± 3.84**	**57.5 ± 5.44**
**2**^**nd**^ **Exercise EE (Kcal)**	**472.5 ± 55.81**	**408.6 ± 24.54**
**Carbohydrate utilization (%) during 2**^**nd**^ **exercise**	**42.0 ± 2.08**^a^	**60.6 ± 3.26**^b^*
**Fat utilization (%) during 2**^**nd**^ **exercise**	**58.0 ± 2.08**^a^	**39.4 ± 3.26**^b^*
**Post-trial resting carbohydrate utilization (%)**	**42.1 ± 12.00**	**44.9 ± 16.35**^#^
**Post-trial resting fat utilization (%)**	**57.9 ± 12.03**	**55.1 ± 16.35**^#^

LCX, exercise trial before the low-carbohydrate meals; HCX, exercise trial before the high-carbohydrate meals; BMI, body mass index; EI, energy intake; EE, energy expenditure; EB, energy balance.

^a,b^ Within the same variable, groups with different superscripts are significantly different (p≤0.05).

* Within the HCX group, more carbohydrate and less fat was utilized during the 2nd than the 1st exercise session (p≤0.05).

^#^ Within the HCS group, less carbohydrate and more fat was utilized during the post- than the pre-trial resting period (p≤0.05).

### Plasma glucose

**Fig 2:**
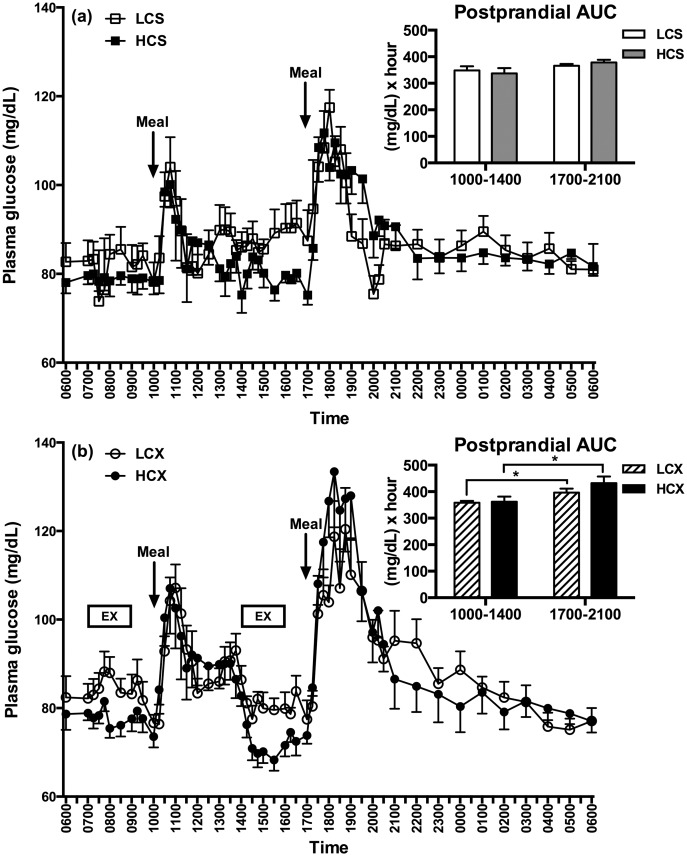
Plasma glucose responses in (a) sedentary and (b) exercise trials with low- and high-carbohydrate meals. LCS, sedentary trial with the low-carbohydrate meals; HCS, sedentary trial with high-carbohydrate meals; LCX, exercise trial with the low-carbohydrate meals; HCX, exercise trial with the high-carbohydrate meals; EX, 2-hour moderate-intensity exercise. *indicates the significant difference (p≤0.05) between the two areas under the curve (AUCs).

Pre-trial fasting plasma glucose was not different in the four experimental groups but tended to be lower after the pre-trial HC than LC meal (Tables 1 and 2). The glycemia after the evening HC meal in the sedentary trials was 12% greater than after the morning meal, and only 5% greater after the evening compared to the morning LC meal (Fig 2a). Thus, evening relative to morning glycemia did not differ in the LCS group, but was higher in the HCS group by parametric statistic (LCS: F = 0.94, p = 0.35, HCS: F = 5.51, p = 0.03), but not by non-parametric test (LC: S = 7, p = 0.38, HC: S = 13, p = 0.08, Fig 2a insert). In contrast to the modest glucose lowering effect of LC diet after the evening meal, exercise exacerbated evening relative to morning postprandial difference after both HC (S = 18, p< 0.01) and LC (S = 17, P = 0.02, Fig 2b insert) meals, and the difference was about twice as great after HC (20%) compared to LC (11%) meals.

### Plasma insulin

**Fig 3:**
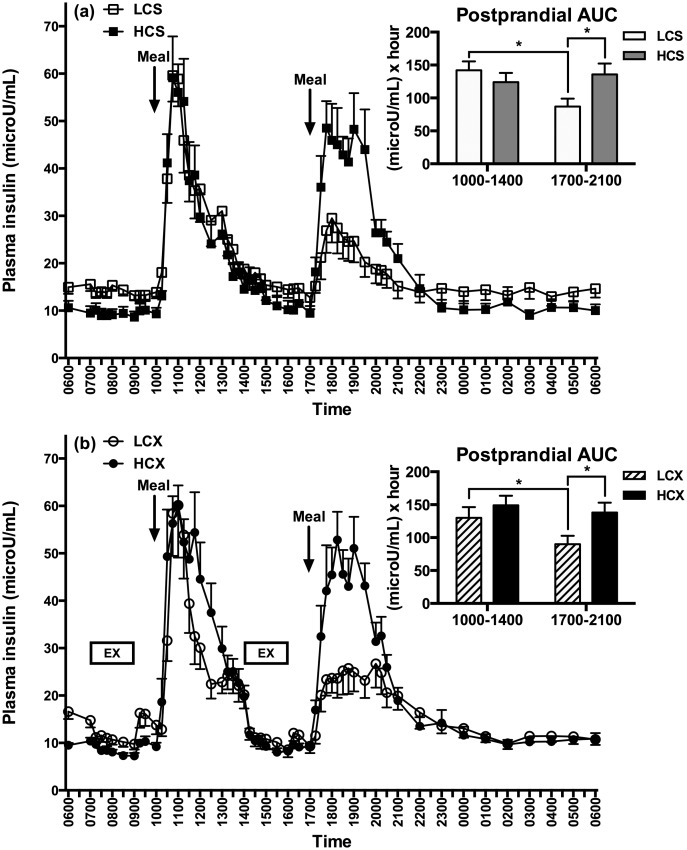
Plasma insulin responses in (a) sedentary and (b) exercise trials with low- and high-carbohydrate meals. LCS, sedentary trial with low-carbohydrate meals; HCS, sedentary trial with high-carbohydrate meals; LCX, exercise trial with the low-carbohydrate meals; HCX, exercise trial with the high-carbohydrate meals; EX, 2-hour moderate-intensity exercise. *indicates the significant difference (p≤0.05) between the two areas under the curve (AUCs).

Fasting plasma insulin at 0600 h in the morning of the sedentary trial day tended to be lower after pre-trial HC compared to LC meals (Table 1) and was significantly lower in the exercise trials (Table 2). Morning postprandial insulin AUCs were equally high after LC and HC meals in both the sedentary (Fig 3a) and the exercise (Fig 3b) trials. However, evening postprandial insulin response to the LC meal was 39% lower than after the morning meal in the sedentary trial (S = 18, p<0.01) and was 31% lower in the exercise trial (S = 18, p<0.01). The evening postprandial insulin response also was 36% lower after the LC than after the HC meals in the sedentary trial (F = 5.93, p = 0.03) and 35% lower in the exercise trial (F = 5.3, p = 0.04). Meal-timing effect was significant in both the sedentary (F = 6.87, p = 0.02) and the exercise (F = 14.39, p<0.01) trials, as was the interaction between the meal timing and the diet composition (sedentary: F = 16.54, p<0.01; exercise: F = 4.67, p = 0.05), but there was no overall dietary effect.

### HOMA-IR estimates of postprandial insulin resistance

**Fig 4:**
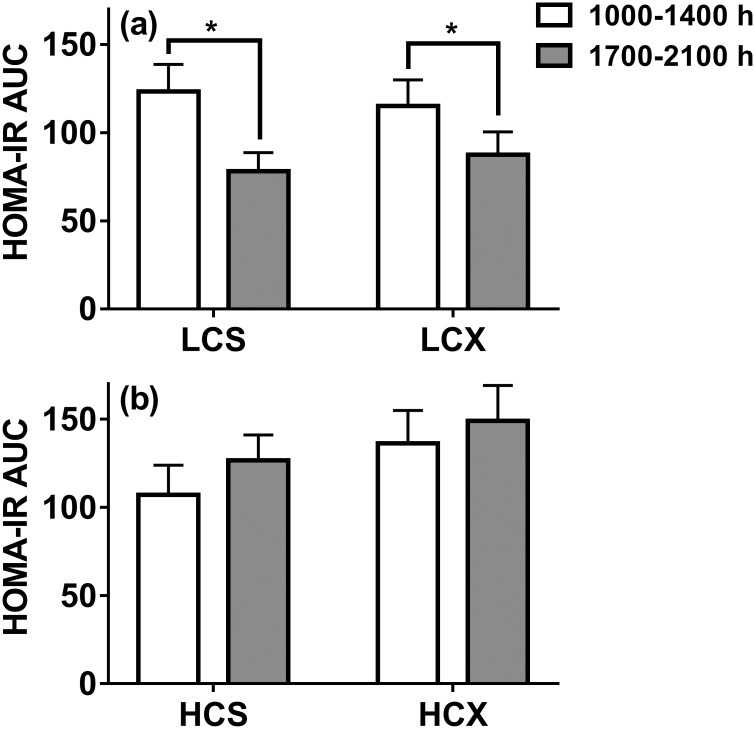
Postprandial HOMA-IR AUCs in (a) low-carbohydrate and (b) high-carbohydrate trials. LCS, sedentary trial with low-carbohydrate meals; LCX, exercise trial with the low-carbohydrate meals; HCS, sedentary trial with high-carbohydrate meals; HCX, exercise trial with the high-carbohydrate meals. *indicates the significant difference (p≤0.05) between two areas under the curve (AUCs).

The HOMA-IR estimate of insulin resistance during the evening postprandial period with the LC diet was significantly lower than during the morning postprandial period in both the sedentary (37%, t = 3.47, p<0.01) and exercise (24%, t = 2.11, p = 0.05) trials (Fig 4a). There was no such difference in the postprandial HOMA-IR scores after the morning and the evening HC meals in either the sedentary or exercise trials (Fig 4b).

### Plasma glucagon

**Fig 5:**
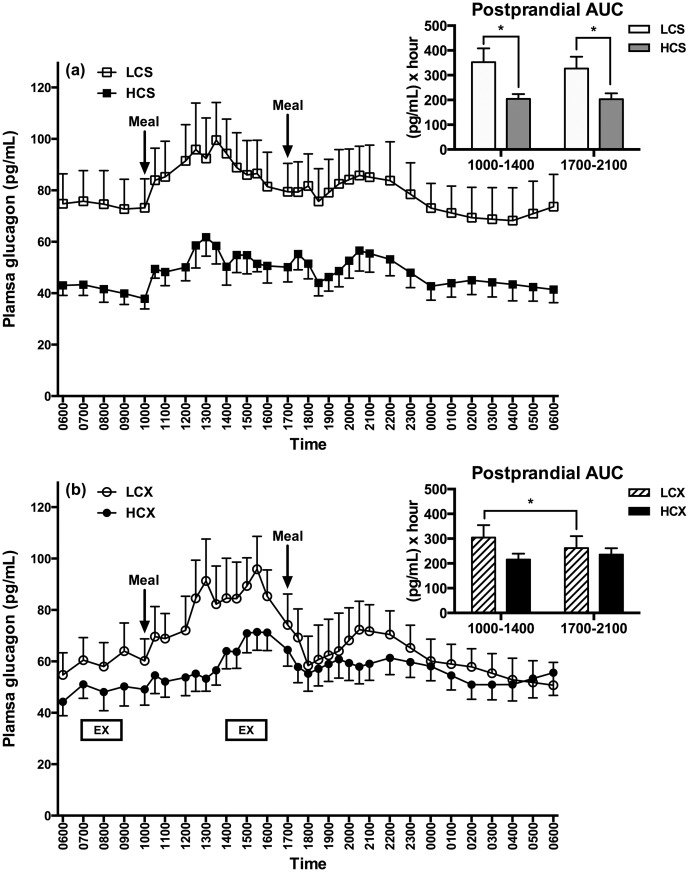
Plasma glucagon responses in (a) sedentary and (b) exercise trials with low- and high-carbohydrate meals. LCS, sedentary trial with low-carbohydrate meals; HCS, sedentary trial with high-carbohydrate meals; LCX, exercise trial with the low-carbohydrate meals; HCX, exercise trial with the high-carbohydrate meals; EX, 2-hour moderate-intensity exercise. *indicates the significant difference (p≤0.05) between the two areas under the curve (AUCs).

In the sedentary trials, postprandial glucagon AUCs after the LC meals were significantly higher in the morning (F = 7.12, p = 0.02) as well as in the evening (F = 4.96, p = 0.04) than after the HCS meals (Fig 5a). In the exercise trials (Fig 5b), postprandial glucagon response to the LC meal was significantly higher in the morning than in the evening (S = 17, p = 0.02, Fig 5b insert), and the postprandial glucagon AUC was significantly higher during the afternoon exercise compared to the morning exercise period in both LCX (S = 18, p<0.01) and HCX (S = 18, p<0.01) groups.

### Plasma GIP

**Fig 6:**
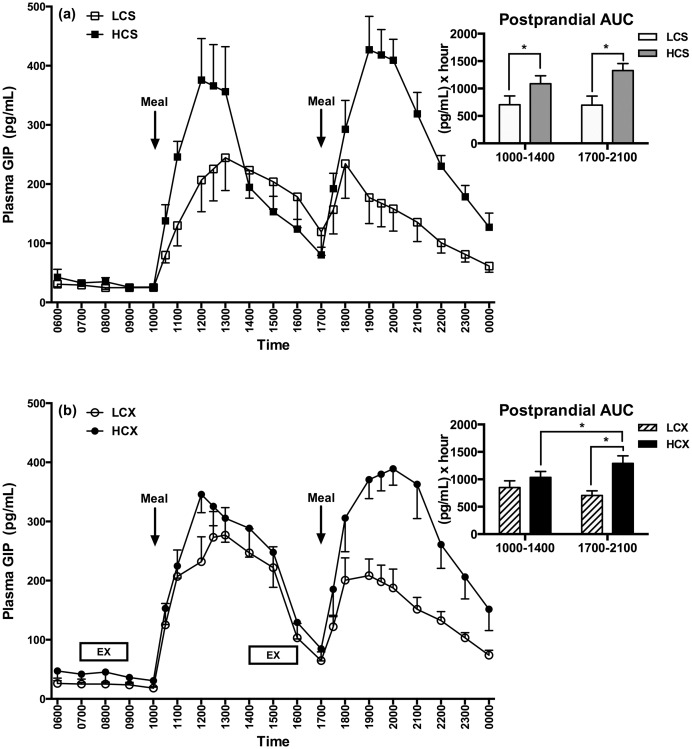
Plasma GIP responses in (a) sedentary and (b) exercise trials with low- and high-carbohydrate meals. LCS, sedentary trial with low-carbohydrate meals; HCS, sedentary trial with high-carbohydrate meals; LCX, exercise trial with the low-carbohydrate meals; HCX, exercise trial with the high-carbohydrate meals; EX, 2-hour moderate-intensity exercise. *indicates the significant difference (p≤0.05) between the two selected time areas under the curve (AUCs).

Exposure to LC diet in the sedentary condition (Fig 6a) reduced the postprandial GIP AUC to a greater extent than the consumption of HC diet after both morning (Z = 1.94, p = 0.05) and evening LC meal (Z = 2.26, p = 0.02). No dietary effect was seen in the morning postprandial GIP responses after the morning pre-meal exercise. However, the evening postprandial response was 45% lower after the LCX than the HCX pre-meal exercise (F = 12.66, p<0.01, Fig 6b). In addition, after the pre-meal exercise, the evening postprandial GIP AUC in response to the HC meal was 25% higher than in the morning (S = 15, p = 0.04, Fig 6b insert). In the exercise trials, postprandial GIP AUC was significantly higher in both LC (S = 18, p<0.01) and HC (S = 18, p<0.01) groups in the afternoon, when exercise took place during the late postprandial period, than when exercise in the morning was in fasted state. A similar difference in GIP AUCs between the corresponding late-postprandial and the fasting periods also was seen in the sedentary trial (LCS: S = 18, p<0.01; HCS: S = 18, p = 0.01).

### Plasma GLP-1

Postprandial GLP-1 responses were not affected by either diet or exercise (data not shown). However, postprandial GLP-1 AUCs were significantly higher during the afternoon, than during the morning exercise in both dietary groups (LCX: S = 18, p<0.01; HCX: S = 18, p<0.01).

### Plasma FFAs

**Fig 7:**
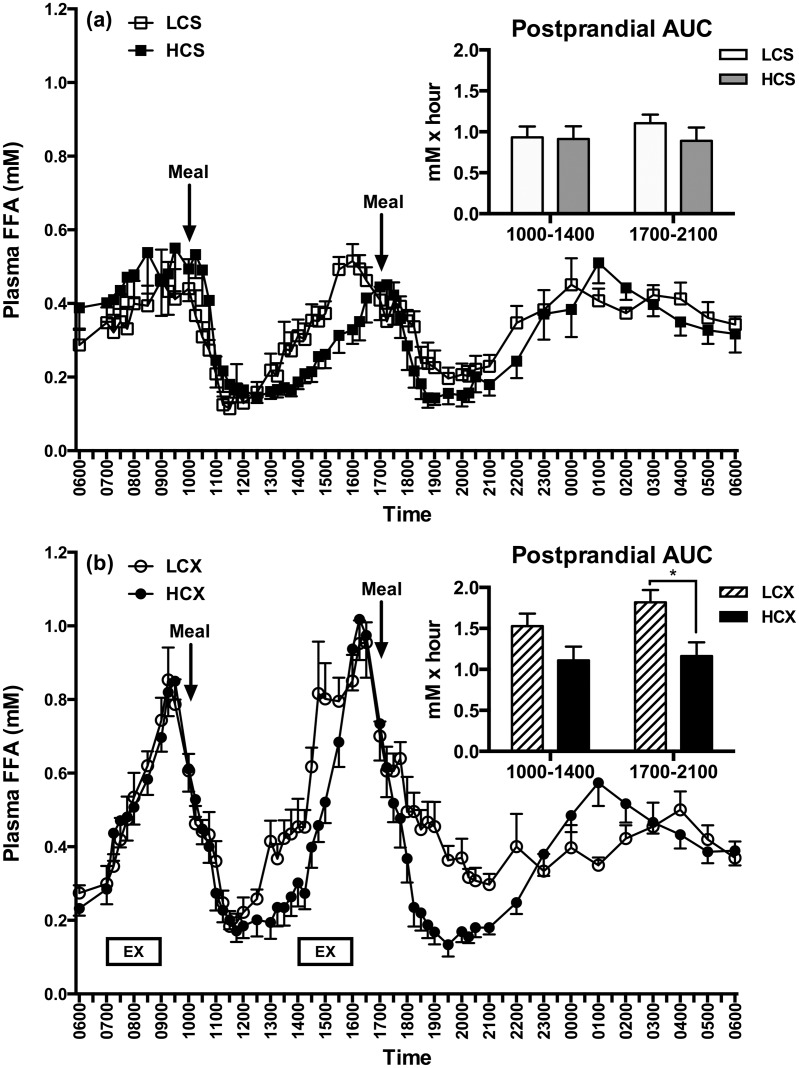
Plasma FFA responses in (a) sedentary and (b) exercise trials with low- and high-carbohydrate meals. LCS, sedentary trial with low-carbohydrate meals; HCS, sedentary trial with high-carbohydrate meals; LCX, exercise trial with the low-carbohydrate meals; HCX, exercise trial with the high-carbohydrate meals; EX, 2-hour moderate-intensity exercise. *indicates the significant difference (p≤0.05) between two areas under the curve (AUCs).

In the sedentary trial, the pre-meal rise in plasma FFAs was greater before the morning than the evening HC meal (F = 6.13, p = 0.03) with no such diurnal difference in LCS group (Fig 7a). In contrast, increase in plasma FFA concentrations during pre-meal exercise was significantly greater in the afternoon than in the morning in both the LCX (F = 10.91, p = 0.01) and the HCX (F = 4.64, p = 0.05) trials (Fig 7b). The evening postprandial FFA AUC also was significantly higher after the LC than the HC meals (F = 8.44, p = 0.01) in the exercise trial (Fig 7b, insert).

### Plasma D-3-hydroxybutyrate

**Fig 8:**
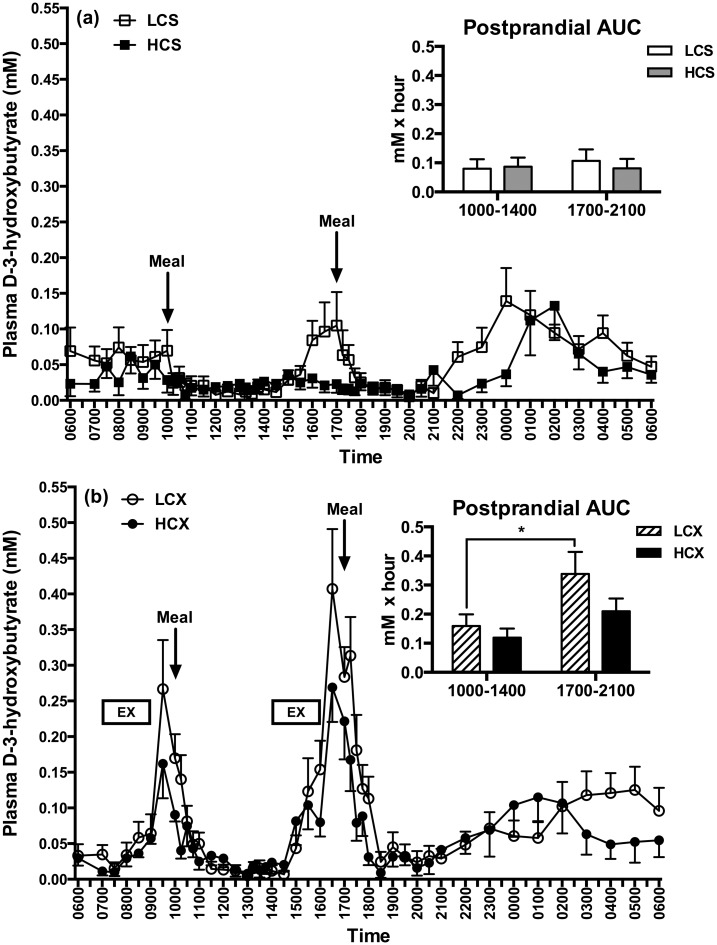
Plasma D-3-hydroxybutyrate responses in (a) sedentary and (b) exercise trials with low- and high-carbohydrate meals. LCS, sedentary trial with low-carbohydrate meals; HCS, sedentary trial with high-carbohydrate meals; LCX, exercise trial with the low-carbohydrate meals; HCX, exercise trial with the high-carbohydrate meals; EX, 2-hour moderate-intensity exercise. *indicates the significant difference (p≤0.05) between two areas under the curve (AUCs).

No timing or dietary effects were seen on the D-3-hydroxybutyrate AUCs in the sedentary trials (Fig 8a). In the exercise trial (Fig 8b), postprandial D-3-hydroxybutyrate AUC was significantly higher after the evening than the morning LC meal (F = 5.83, p = 0.03). Plasma D-3-hydroxybutyrate AUCs during pre-meal exercise periods were significantly higher in the afternoon than in the morning in both the LCX (F = 5.05, p = 0.04) and the HCX (F = 5.2, p = 0.04) groups (Fig 8b).

## Discussion

The principal significant finding of this study was the observation of parallel declines in evening postprandial insulin and GIP responses with a concurrent decline in postprandial insulin resistance as estimated by HOMA-IR after the third exposure to the low-carbohydrate meal in metabolically healthy individuals with normal glucose tolerance. This outcome was unaffected by pre-meal exercise and absent after the high-carbohydrate meals. The remarkable features of this finding were that postprandial insulin and GIP declines to the low-carbohydrate, relative to the high-carbohydrate, meals were strikingly similar both in their magnitude under both the sedentary and the exercise conditions (39% and 31% declines in insulin and 48% and 45% declines in GIP, respectively) and that they also were synchronous in the timing of their decline. A significant decline in the postprandial GIP AUC after the morning meal preceded the simultaneous declines in both the postprandial GIP and insulin responses after the third low-carbohydrate meal in the evening suggesting a probable causative role of the GIP incretin decline in the evening postprandial insulin decline. This observation prompts two tentative conclusions.

The first one is that the decline in the evening postprandial insulin response and in the estimate of insulin resistance after a 24-hour exposure to low-carbohydrate meals is driven by the intestinal processing of carbohydrates rather than by any changes in muscle glucose uptake as the latter would have been affected by pre-meal exercise. The likely sequence of effects produced by a third exposure to a low-carbohydrate meal is sensing of reduced glucose concentration in the proximal brush-border epithelium by the small intestinal glucose sensor, the sodium-glucose co-transporter-1 (SGLT1) [44,45]. In contrast to SGLT2 which mediates glucose reuptake in the kidney cortex [44], the inhibition of which is used in the treatment of diabetic hyperglycemia [46], SGLT1 senses and mediates glucose absorption and stimulates GIP secretion in the upper intestine. Thus reduced activation of SGLT1 by a one-day of exposure to a low-carbohydrate diet is likely to have reduced the stimulation of GIP release [47], which then may have led to a proportional reduction in its postprandial insulinotropic action [48]. By contrast, postprandial GIP and insulin responses to morning and evening high-carbohydrate meals remained high, and the evening postprandial GIP response was even 25% higher than the morning one.

Our second tentative conclusion is that a 24-hour delay associated with three exposures to low-carbohydrate diet in the lowering of postprandial insulin response, most likely reflects the latency in the intestinal adjustment to the changes in the carbohydrate load. Although we did not investigate the type of diets habitually eaten by the 32 subjects in our study, it is highly probable that it approximated the 51% dietary carbohydrate content reported for the US population in 2011 [11]. The apparent requirement for the intake of three successive low-carbohydrate meals over a 24-hour period to produce a decline in the evening postprandial insulin response may represent the length of time necessary for an adjustment in GIP secretion to the intestinal processing of the reduced carbohydrate load. The 24-hour delay in downward adjustment in postprandial insulin response and insulin resistance to a reduced carbohydrate meal in this study is analogous to the three-day requirement for the upward adjustment in insulin sensitivity in response to 250- to 300-gram intake of carbohydrates (known as Staub-Traugott effect) customarily recommended and used before tests of fasting insulin sensitivity [49]. Both phenomena may reflect a necessary time course in intestinal and endocrine adjustments to changes in dietary carbohydrate.

The significant reduction in the evening postprandial insulin response and HOMA-IR estimate of insulin resistance after a day’s exposure to low-carbohydrate meals, raises two questions about the contribution of daily carbohydrate load to this effect. The first one addresses the absolute size of carbohydrate load responsible for the changes in the evening postprandial insulin response, and insulin resistance. Reducing acute dietary carbohydrate exposure in three steps by a factor of two, from 125 to 31.25 g of carbohydrate per meal using a 50% carbohydrate diet, abolished evening hyperglycemia with 31.25 g of carbohydrate per meal or a with total daily carbohydrate load of 95 g [21]. At 62.5 g of carbohydrate per meal and a daily carbohydrate load of 187.5 g, evening postprandial glycemia increased 2.8-fold, and at 125 g of carbohydrate per meal and 375 g per day, it increased 3.5-fold. The interpretation of these data is somewhat confounded by the concomitant manipulation of the energy content of the meals which also was reduced in three steps by a factor of two, from 50% to 12.5% of the weight-maintenance amount, as this also could have influenced postprandial insulin response. However, these data concur with ours that a daily carbohydrate load of between 95 and 115 g can acutely reduce high evening postprandial glycemia and insulin response. That amount of daily carbohydrate would contribute about 25% of calories to a 1600 Kcal diet.

The second question addresses the influence of the timing of exposure to high carbohydrate load on the evening postprandial glycemic and insulin responses. This is relevant to our study since we offered two relatively large iso-caloric 740 to 820 Kcal meals rather than the customary three smaller daily meals. Two and a half-fold higher evening compared to morning postprandial glycemia after the high-carbohydrate relative to low-carbohydrate meals (12.3% vs 4.9%) may have been influenced by delivery of 120 g as opposed to 58 g of carbohydrate in the evening meal. Greater carbohydrate loads in the evening than in the morning were shown to produce evening glucose intolerance is several studies. When 60% of daily calories and 181 g of carbohydrate in a 54.5% carbohydrate diet were eaten at dinnertime, evening postprandial glycemia was 20% greater than when this large meal was eaten at breakfast [20]. Similarly in individuals with T2D, postprandial glycemia was 20%, and insulin 11%, lower after the three daily meals when 700 kilocalories were eaten in the morning and a 204 Kcal meal in the evening than when the sizes of the meals were reversed [50]. Additional evidence for a circadian influence on the capacity of the pancreas to secrete insulin was provided by a study where a morning and an evening iso-caloric meal containing a daily 225 to 250 Kcal carbohydrate load were eaten after adaptation to either normal or reversed 12-h light-dark photoperiods [19]. A circadian influence accounted for 12% of the evening glucose intolerance that was attributed to a 27% reduction in early-phase insulin secretion. Under reversed day-night regime in the absence of circadian influence, daily exposure to the dietary carbohydrate load contributed another 8% to the evening postprandial hyperglycemia, this time attributable to a decrease in both β cell secretory function and to peripheral insulin resistance. Reduced capacity of pancreas to secrete insulin or clear glucose when a large amount of carbohydrates is eaten later in the day suggested by this study [19] is also supported by the requirement for a higher evening dose of insulin secretagogue tolbutamide [13] and of insulin in type 1 diabetics [18]. However, evening glucose intolerance in all of these studies may be related to the high total daily carbohydrate content of the meals.

Presence of high evening postprandial insulin response, and HOMA-IR score in our study during acute one-day exposure to 60% carbohydrate diet in metabolically healthy, glucose tolerant individuals raises the question of whether this effect may become clinically significant with extended exposure to this diet in view of the health risks of chronic hyperglycemia and hyperinsulinemia of Type 2 diabetes [2–6]. This question was addressed in studies which examined the effects of five days [51] and two weeks [52] of adaptation to diets differing in carbohydrate content on fasting insulin and glucose, fasting hepatic glucose production (HGP), and fasting metabolic fuel use. A 2723-kcal reference diet containing 47% of carbohydrate energy was manipulated by either adding or removing 25% and 50% of carbohydrates generating at the extremes of this range a dietary macronutrient composition that was similar to our low-carbohydrate and high-carbohydrate diets [51]. Removal of 50% of carbohydrates produced a 32% carbohydrate, 47% fat, 21% protein diet, and adding 50% of carbohydrates changed the dietary composition to 59% carbohydrate, 29% fat, and 12% protein. Increasing the 5-day carbohydrate intake from 114g to 684 g doubled the fasting insulin concentration, increased fasting HGP by 41%, and reduced whole-body lipolysis by 52% and fat oxidation by 91%. As the fasting glucose concentration remained unchanged, proportional increases in fasting insulin to a 5-day adaptation to a higher dietary carbohydrate load increased fasting HOMA-IR twofold. Although this manipulation also altered daily energy intake from about 1400 to 4500 Kcal, a doubling of fat calories to 57% and a comparable increase of total daily energy intake to 4200 Kcal resulted in fasting glucose, insulin, and HOMA-IR as low as with a daily intake of 114 g of carbohydrates and 1400 Kcal, and fat oxidation and hepatic glucose production unchanged from the reference diet. Similar 26% and 33.5% increases, respectively, in fasting insulin and HOMA-IR were observed in metabolically healthy subjects after two weeks of adaptation to a 75%-carbohydrate compared to a 55%-carbohydrate diet [52]. These two studies suggest that current habitual consumption of 51%-carbohydrate diets in the US [11] may increase fasting insulin resistance in a clinically relevant way and promote body fat accumulation by reducing lipolysis and fat oxidation through stimulation by the high postprandial insulin concentrations of the adipose tissue lipoprotein lipase [53]. Our study using acute one-day alteration of dietary carbohydrate content and the two studies extending it to between 5 [51] and 14 days [52] suggest that a reduction of carbohydrate content of the meals from about 60% to about 30% of daily energy may substantially reduce this potential risk.

Pre-meal exercise exacerbated evening glucose intolerance under both dietary conditions. At 45% of maximal effort, lipid utilization contributed between 57 and 59% of energy for pre-meal exercise in both of our dietary groups as was also documented by others [54]. We observed significant increases in plasma concentrations of free fatty acids during both morning and afternoon pre-meal exercise bouts that attained near-peak levels at the start of the meals. This may have contributed to greater postprandial glycemia in exercise trials as muscle uptake of free fatty acids interferes with insulin signaling [39]. In addition, it is likely that residual increased endogenous glucose production during pre-meal exercise [26,27] compounded the contribution of meal-associated glucose to circulation. Exercise-induced activation of endogenous glucose production and lipid substrate utilization are the likely reason why exercise before the meals in fasted or post-absorptive state consistently fails to improve glucose tolerance [55–57] while exercise during the early postprandial period improves it [56–59]. It is noteworthy that the exacerbation of postprandial glycemia by pre-meal exercise has taken place in spite of our selection of the exercise intensity of 45% of maximal effort and 2-hour long bouts of exercise, both conducive to facilitation of post-exercise insulin sensitivity. Insulin sensitivity and glucose tolerance increase at exercise intensities in the range of between 35% and 55% of VO_2_ max [60–62] but not at intensities above 60% of VO_2_ max [62,63]. Each two-hour exercise bout in our study expended about 450 Kcal, exceeding the 300-Kcal exercise energy expenditure necessary to increase insulin sensitivity several hours after its termination [63,64].

For an explanation of high HOMA-IR scores during the morning postprandial periods after both LC and HC diets, and during the second postprandial period after HC meals, it is useful to view the fasted or post-absorptive states as conditions of muscle fuel repletion and of shortage of circulating fuel. At the start of the morning postprandial period, fasting muscle is glycogen and lipid replete [65] and associated with insulin resistance [60, 66]. In addition, absence of absorbed nutrient entry into the circulation during fast requires mobilization of the storage fuels by counter-regulatory hormones that block glucose utilization by peripheral tissues and raise systemic insulin resistance. Increased glucose availability provided by the second high-carbohydrate meal also is likely to have increased muscle glycogen synthesis and further contributed to insulin resistance [66].

This study has several limitations. We did not directly measure peripheral glucose uptake or insulin sensitivity by the intravenous glucose tolerance test or by the hyperinsulinemic euglycemic clamp. Despite their established advantages in assessing insulin sensitivity, these methods bypass gastrointestinal route of carbohydrate intake and require testing of the effects of experimental manipulations with identical glucose and insulin challenges. This made them unsuitable for the assessment of changes in postprandial glycemia to oral intake of meals differing in carbohydrate content. We chose the HOMA-IR estimate of insulin resistance during the meal tolerance tests in part because it allows assessment of the physiological contribution of intestinal incretins to insulin secretion. This is a surrogate test for the intravenous frequently-sampled glucose tolerance test against which it has been validated [42, 43]. The model assumes that the systemic glucose disposal equals glucose entering the circulation and therefore should be applied, as was the case in this study, to comparisons between the meals that have the same carbohydrate content [67]. We used only metabolically healthy postmenopausal women in this study, so the results may not be generalizable to individuals of different age, gender, or levels of insulin resistance. We did not expose our subjects to pre-trial dietary and exercise standardization, but relied on their normal fasting glucose and uniform health statistics and aerobic fitness as sufficient evidence of adequate matching as is the case in a number of studies [20]. We did not make direct assessment of muscle glucose uptake or muscle glycogen turnover to evaluate contribution of changes in peripheral glucose uptake to dietary or exercise manipulations. Finally, we opted to provide daily weight-maintenance energy intake within two large iso-caloric meals that may have affected evening postprandial glycemia and insulin responses. Although the meals were iso-caloric and of identical macronutrient composition, they did not contain identical food items as human habitual eating behavior entails consuming different food in the morning and in the evening. In addition, the glycemic index (GI) of the meals was not identical, with the evening high-carbohydrate meal being about 26% higher than the other three meals which differed only by between 2 and 7%. Since the decline in the postprandial GIP and insulin that occurred with low-carbohydrate meals had GIs that differed by only 2%, and the morning postprandial GIP and insulin responses after both diets were high despite an almost identical GI, the GI difference in the meals may have had only a minor, if any, effect on the glycemic differences associated with diets differing in carbohydrate content. Two and a half-fold difference in GI was necessary to produce significant effects on postprandial glycemia [20]. Our LC and HC meals did not have identical sugar to non-sugar carbohydrate ratios as was the case is some other studies [52]. However, it is unlikely that the slightly higher sugar to non-sugar carbohydrate ratio of 52:48 in the three low-carbohydrate meals compared to 43:57 in high-carbohydrate meals had a significant endocrine and metabolic consequences since we obtained a reduction in evening postprandial HOMA-IR after the former and not after the latter.

Despite these limitations, our results and consideration of the work of others allow us to make the following conclusions: First, a third successive meal containing about 57 g of carbohydrates and contributing to a cumulative daily carbohydrate load of about 115 g, significantly reduces evening postprandial insulin and GIP responses and HOMA-IR estimate of insulin resistance, while the same number of iso-caloric meals containing about 120 g of carbohydrates and a daily carbohydrate load of 241 g contributes to evening postprandial hyperinsulinemia and a high HOMA-IR score. Second, the parallel three-meal or 24-hour delay in the onset, and a similarity in the magnitude, of declines in postprandial insulin and GIP responses suggests their probable interdependent responses to a low-carbohydrate diet. The three-meal delay in the reduction of the evening postprandial insulin and GIP response appears to represent a delay in the adaptation of intestinal glucose sensing to the intake of low-carbohydrate meals. Third, the decline in the evening postprandial insulin, GIP, and HOMA-IR to third exposure to a low-carbohydrate meal is a dietary effect unaffected by any changes in muscle glucose uptake during pre-meal exercise. In effect, pre-meal moderate-intensity exercise exacerbates evening glucose intolerance regardless of the dietary composition of the meals and without an effect on the dietary influence over evening postprandial insulin, GIP, and HOMA-IR. The exercise effect is most likely mediated through its stimulation of endogenous glucose production [26,27] compounding the postprandial addition of absorbed dietary glucose and through the interference by mobilized free fatty acids with insulin signaling in the muscle. By demonstrating that a three successive-meal exposure over a 24-hour period to a different dietary carbohydrate content controls evening postprandial hyperinsulinemia and HOMA-IR, we provide an experimental model for the study of the question whether the protracted exposure to diets differing in carbohydrate content may affect the long-term risk for insulin resistance and pre-diabetes, as was suggested by studies with diabetic subjects [38].

## Supporting Information

S1 Checklist TREND statement checklist. (PDF)

S1 Protocol Detailed IRB clinical trial protocols. (PDF)
