# Supplementary material for: Third Exposure to a Reduced Carbohydrate Meal Lowers Evening Postprandial Insulin and GIP Responses and HOMA-IR Estimate of Insulin Resistance
Source: PLoS One. 2016 Oct 31;11(10):e0165378. doi: 10.1371/journal.pone.0165378 (PMC5087910; doi:10.1371/journal.pone.0165378)
Supplement: S1 Protocol — (PDF) [file pone.0165378.s002.pdf]

Research protocol for Timing of exercise and meals for prevention of type 2 diabetes (HUM00017875)

ABSTRACT

Two spaced bouts of moderate-intensity exercise reduce counterregulatory responses to a next-day hypoglycemic challenge and lead to hypoglycemia, a phenomenon that was characterized as exercise-associated autonomic failure (EAAF). We have recently found that a 19% lowering of blood glucose and altered pancreatic hormone response depend on exercise being performed in fasted/postabsorptive state, as none of the changes occurs when exercise is carried out in post-prandial state. We therefore think that inadequate hepatic glycogen recovery due to preferential muscle glucose uptake during and after exercise, rather than autonomic failure, is responsible for EAAF. The magnitude of the effect achieved in our preliminary study was comparable to that produced by 12 weeks of administration of 850 mg of metformin twice daily (19% reduction) or 45 mg of pioglitazone (22 % reduction) (1), without the side effects associated with the drugs and with added benefits of exercise.

__Research Objectives__:

(1) Determine whether exercise before the meals has the blood-glucose lowering effect because of reduced hepatic glucose production and increased exercise and post-exercise muscle glucose uptake. We will measure hepatic glucose production and muscle glucose uptake during heavy glucose isotope infusion. Our hypothesis is that exercise before meals results in inadequate liver glycogen recovery, as manifested by increased ketone body production, due to increased exercise and post-exercise muscle glucose uptake.

(2) Assess whether a 50% reduction of the exercise volume before meals (to one hour twice daily) coupled with a 50% reduction in carbohydrate content of meals (to 30% carbohydrate, 25% protein and 45% fat) will be as effective in lowering plasma glucose as the original paradigm (2 hours of exercise twice daily and meal macronutrient content of 60% carbohydrate, 15% protein, and 25% fat). Our hypothesis is that muscle glucose uptake following two spaced one-hour exercise bouts combined with a 50% lower carbohydrate content of the diet, will be of sufficient magnitude to produce inadequate hepatic glycogen repletion, reduced hepatic glucose production, and sustained lowering of blood glucose.

The novel contribution of this research is that it will provide a scientific rationale for prescription of effective exercise and diet protocols for prevention of type 2 diabetes.

PROPOSAL NARRATIVE

__A. SPECIFIC AIMS__

__**Specific aim 1**__:

Determine whether exercise before the meals has blood-glucose lowering effect because of reduced hepatic glucose production and increased exercise and post-exercise muscle glucose uptake. We will measure (a) concentrations of glucose, ketone bodies, insulin, and glucagon with appropriate spectrophotometric and radioimmuno assays, (b) total body glucose uptake and (c) the rate of hepatic glucose production with stable glucose isotope infusion and mass spectrometer, and (d) substrate utilization with indirect calorimetry, in three trials: two bouts of moderate-intensity exercise each producing expenditure of 500 Kcal and separated by 5 hours, performed either before the high-carbohydrate meals or after the meals, and a sedentary control trial.

__**Hypothesis 1**__: Two spaced bouts of moderate-intensity exercise, when performed before the high-carbohydrate meals, but not after such meals, will (a) increase muscle glucose uptake during and following exercise, (b) result in inadequate liver glycogen repletion as manifested by high ketone body

production, (c) reduce hepatic glucose output, and (d) produce sustained reduction in basal plasma glucose level.

<u>Specific aim 2:</u> Determine whether a modification of the original experimental paradigm that is likely to produce easier protocol adherence will have the same glucose lowering effect. We will assess whether a 50% reduction of the exercise volume before the meals (to one hour twice daily) coupled with a 50% reduction in carbohydrate content of the meals (to 30% carbohydrate, 25% protein and 45% fat) will be as effective in lowering basal plasma glucose as the original paradigm (2 hours of exercise twice daily and meal macronutrient content of 60% carbohydrate, 15% protein, and 25% fat). We will measure (a) concentrations of glucose, ketone bodies, insulin, and glucagon with appropriate spectrophotometric and radioimmuno assays, (b) total body glucose uptake and (c) the rate of hepatic glucose production with stable heavy glucose isotope infusion and mass spectrometer, and (d) substrate utilization with indirect calorimetry, in the fourth trial.

<u>Hypothesis 2</u>: Muscle glucose uptake following two spaced one-hour exercise bouts combined with a low carbohydrate content of the diet, will be sufficient to produce inadequate hepatic glycogen repletion, reduced hepatic glucose production, and sustained lowering of blood glucose.

B. <u>BACKGROUND AND SIGNIFICANCE</u>

Because of the damage to peripheral organs that results from chronic hyperglycemia, normalization of blood glucose levels is an important goal for treatment of diabetes. Diet and exercise are the mainstay for the treatment and even prevention of type 2 diabetes (2). However, appropriate diet and exercise prescription has not been clearly defined, and current approach to new onset type 2 diabetes is pharmacological treatment aimed at reducing persistent hyperglycemia through enhancement of insulin secretion, suppression of elevated hepatic glucose production, or increase in peripheral insulin sensitivity.

Release of glucose into circulation by the liver is under neuroendocrine regulation by insulin and counterregulation by glucagon, catecholamines and several other hormones which insures relative stability of circulating glucose concentration and supply during rest and short-term exercise. Glucose level is maintained during one to two hours of continuous moderate-intensity exercise despite increased insulin-independent muscle glucose uptake (3). Plasma glucose gradually declines toward the hypoglycemic level after four hours of continuous low-intensity exercise (4) or three hours of moderate-intensity exercise (5), despite robust increases in counterregulatory response. This likely reflects a limit in hepatic glucose production capacity rather than an altered neuroendocrine response, as supplying glucose orally (6) or intravenously (7, 8) normalizes plasma glucose during prolonged exercise, and appropriately suppresses counterregulatory hormone levels.

Glucoregulation is altered when moderate-intensity exercise of 90-minute duration is repeated after 3 hours of rest. This treatment, labeled exercise-associated autonomic failure (EAAF, 9), reduces or eliminates counterregulatory response (as well pancreatic polypeptide release, a marker of parasympathetic activity), to a hypoglycemic challenge administered six to 24 hours later (10-14) and has been used as a model for study of exercise-associated complications in type 1 diabetes. In EAAF, hepatic glucose output declines, and plasma glucose concentration is reduced toward hypoglycemic levels in both healthy (10-12) and type 1 diabetic subjects (13,14) several hours to 24 hours after spaced exercise. An interval of several hours between two exercise bouts in combination with a 10-h fast at the outset, appear to be necessary for altered glucoregulation as neither continuous exercise in a severely energy deficient state following a 72-h fast (15), or repeated exercise bouts of 30-min duration separated by shorter 30-minute rest periods (16), suppress vigorous counterregulatory response.

For effective exercise prescription to prevent type 2 diabetes, it is important to understand the mechanism of exercise-associated lowering of blood glucose. I hypothesized that sustained hypoglycemia observed

several hours to a day after two spaced bouts of exercise is due to inadequate hepatic glycogen recovery, rather than autonomic failure, and is caused by increased exercise and post-exercise muscle glucose uptake when exercise in carried out in fasted or postabsorptive state. Increased muscle glucose uptake during exercise is proportional to exercise intensity (17), contributes about one half of increased energy utilization at moderate exercise intensities (18), and is mediated by non-insulin dependent glucose transport (2). After exercise, sustained increases in glucose uptake by the muscle result from an acute increase in muscle insulin sensitivity (19, 20), activation of glycogen synthase in proportion to the level of muscle glycogen depletion (21), and phosphorylation and intramuscular sequestration of glucose at hypoglycemic glucose concentrations by the enzyme hexokinase so long as glucose is metabolized for fuel or for glycogen synthesis (22). As a result, muscle glucose uptake and glycogen resynthesis take precedence over liver glucose uptake and glycogen resynthesis for between several hours (23) and up to three days after glycogen depleting exercise, allowing not only for compensation but also for supercompensation of muscle glycogen stores (24).

C. PRELIMINARY STUDIES

In a recently completed study (25), 9 postmenopausal women (age 58.5 +/- 1.7 years, body mass 74.9 +/- 4.3 kg, BMI 27.0 +/- 1.4 kg/m^2, and 37.3 +/- 2.7 percent body fat) expended 350 Kcal twice in a day while walking 2 h in the morning and 2 h in the afternoon, on one occasion before meals (available at 10 and 17 h) , and on another occasion 1 h after the meals. We found that:

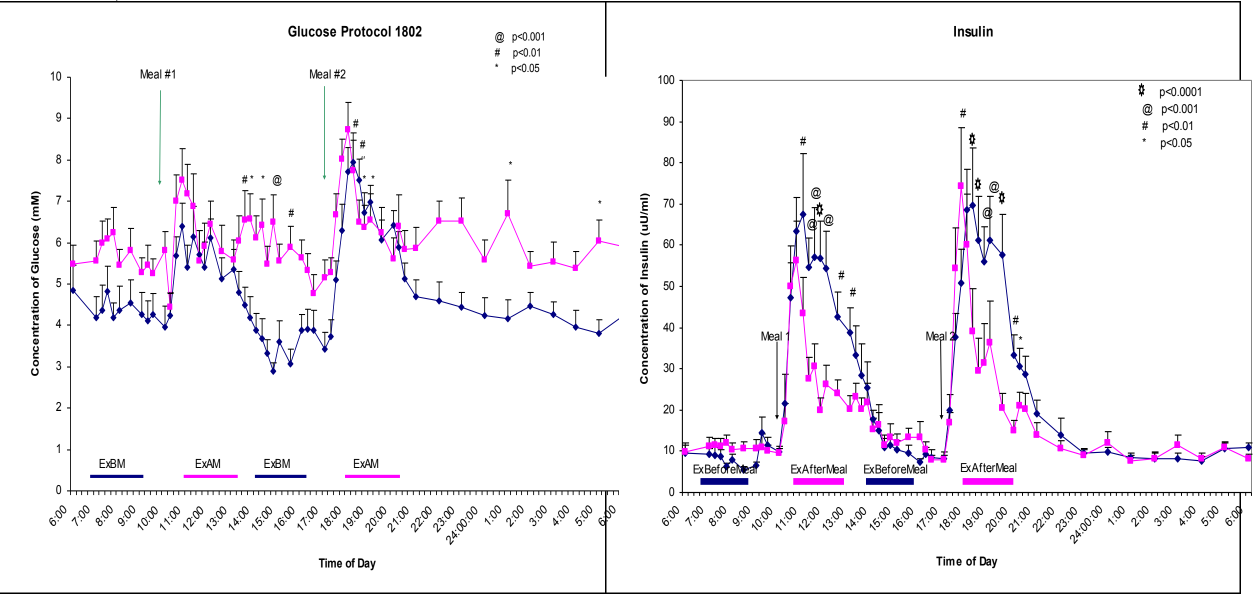

Figure 1 (Left): The 19% decline in basal plasma glucose concentration following the post-exercise meals in before-meals trial was sustained throughout the night.

Figure 1 (Right): Post-meal plasma insulin AUCs were about 50% higher in before-meals trial compared to after-meal exercise trial.

Basal plasma glucose concentration was reduced by 19% during a 16-h period following the post-exercise meals (Figure 1, left) while post-meal plasma insulin areas under the curve (AUCs) were significantly higher (Figure 1 left) in before-meals exercise trials compared to after-meals exercise trials.

In contrast to reduced plasma glucose and increased post-meal insulin response in the before-meals exercise trials, insulin to glucagon ratio was twice as high (Figure 2, left) and ketone body concentration was significantly increased (Figure 2, right) in before-meals, compared to after-meals trial. Plasma concentrations of counter-regulatory hormones glucagon, growth hormone, and cortisol increased during exercise, but did not differ between the two trials (data not shown).

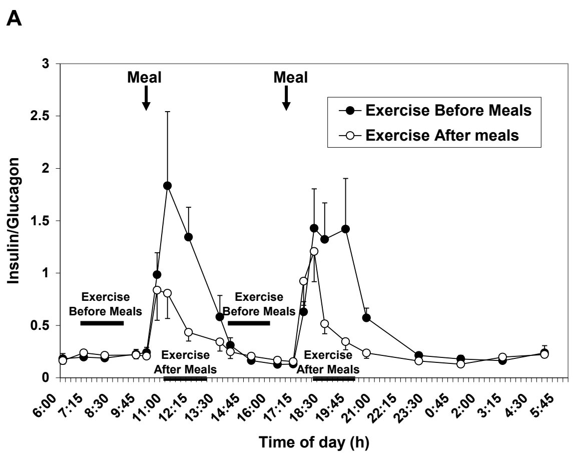

Figure 2 (Left): Insulin-glucagon ratio is about twi higher in the Before-Meal compared to After-Meal trial

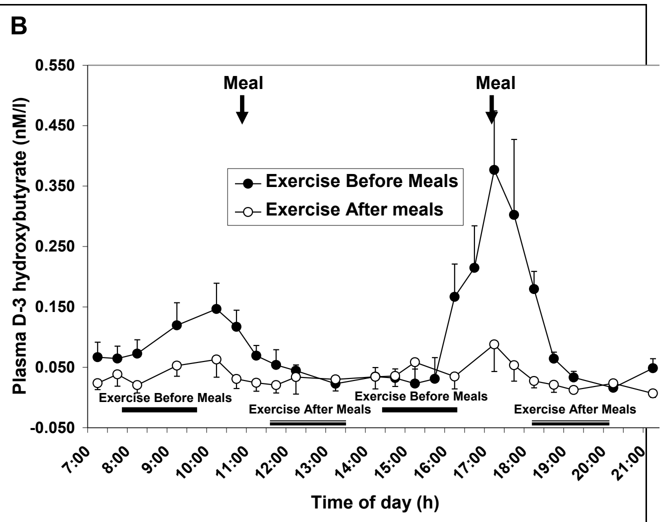

Figure 2 (Right): Post-exercise ketosis develops in th Before-Meal but not in the After-Meal trial indicating hepatic glycogen depletion

Thus these preliminary data demonstrate that two exercise bouts separated by several hours do not inevitably produce delayed "EAAF" hypoglycemia. Instead, sustained lowering of blood glucose is contingent on exercise being performed in fasted or postabsorptive state. Development of post-exercise ketosis demonstrates hepatic carbohydrate depletion which can be terminated by ingestion of glucose or alanine and prevented by prior intake of carbohydrate rich diet (26-29). We hypothesize that increased glucose uptake by the muscle during and after exercise prevents liver glycogen recovery over an extended period of time, as has been shown previously. This condition then leads to reduced basal plasma glucose either through change in neuroendocrine regulation or through protracted dominance of muscle glucose uptake for glycogen repletion.

D. RESEARCH DESIGN AND METHODS

Subjects

31 postmenopausal nondiabetic women of similar characteristics as in original study will be recruited. All subjects will be screened with a detailed history and physical examination, routine blood tests including fasting plasma glucose and insulin, resting metabolic rate measured by indirect calorimetry (V-Max, Viasys, Cardinal Health, Palm Springs, CA), body weight, height, and body composition analysis by dual-energy x-ray absorptiometry (DXA; this body composition scan has been included in the screening tests for the first 20 women and it will be postponed and scheduled during subject's first study trial for the last 11 women.), and a TSH measurement for normal thyroid function. Subject fasting plasma glucose will be less than 100 mg/dl. The exclusion criteria will be metabolic disease and prescription medications other than for hypothyroidism. All subjects will sign an informed consent document approved by the University of Michigan Medical School Institutional Review Board.

- Studies will be performed in the Michigan Clinical Research Unit (MCRU) where subjects will be admitted at 18 h before the study day and receive a standard evening meal at 19 h containing 60% carbohydrate, 15% protein, and 25% fat. The energy content of the meal will be 30 Kcal/kg current body weight and total calories will be one third of daily amount. Each hospital stay during an individual trial will last 36 hrs for the first 18 women but will last 40 hrs for the last 13 women.

Subjects will be assigned in random order to two trials pertaining to hypothesis 1. The two trials will be separated by a week. The order in which any particular treatment pair is implemented, will be balanced.

General study design (Figure 3)

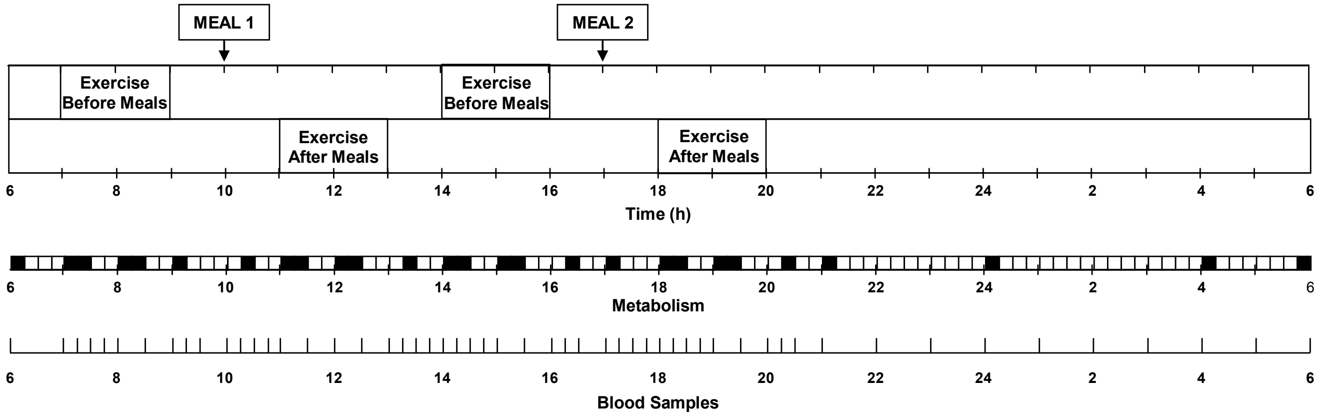

- In 17 women, two catheters will be inserted into arm veins at 19:30 h before the study day in study 1, one for blood collection and the other for isotope infusion. In the 18th woman, one catheter will be inserted at 19:30 h before the study day (Day 1) and the other, in the contralateral vein, at 19:30 h on the day of the study (Day 2). In the last 13 women, the second catheter for isotope infusion will not be inserted until 20:00 h on Day 2, in the contralateral vein. Peak aerobic capacity of the subject will be determined within a week before the start of the study from a screening treadmill test. This test involves collection of ventilatory and respirometric measurements from the subject during progressive increases in treadmill slope (2% every 3 minutes) while walking on a level treadmill at 3 mph. During this screening test, as well as during the exercise portion of the study, the subject will breathe through a mouthpiece equipped with a two-way valve while the rate of ventilation, oxygen consumption (VO_2) and carbon dioxide production (VCO_2) are measured by indirect calorimetry. The results of this test will allow us to determine the walking speed at 50 % of subject's peak effort for 10 subjects in the study, 40% of peak effort for another 10 subjects, and 45% for the last 11 subjects in the study. Energy expenditure of exercise will be measured for 30 min at hourly intervals from oxygen (O_2) and carbon dioxide (CO_2) concentration t$_h$at are continuously sampled from a mixing-chamber and analyzed by the Physio-Dyne metabolic cart (Quogue, NY). The VO_2 and the respiratory quotient (ratio of VCO_2 over VO_2) obtained allow the calculation of calories expended during exercise and the contribution of carbohydrates and lipids to the energy expenditure (17, 29).
- Resting metabolic rate (RMR) will be obtained with V-Max respiratory gas apparatus (Viasys, Cardinal Health, Palm Springs, CA) between 6 and 6:30 h on the day of the experiment and at the same time on the morning after the completion of the study. RMR is assessed with the help of a canopy placed over subject's head, allowing measurements of breathing rate and respiratory gas analysis.
- Meals will be provided at 10 and 17 h. Macronutrient composition of meals in study 1 will be 60% carbohydrate, 15% protein, and 25% fat. Energy content of the meals will be 25 Kcal/kg body weight and each meal will provide one half of the daily amount. We will also provide 20ml of oral liquid acetaminophen (160mg/5ml), which has been used as a marker drug to study the rate of gastric emptying (30), 30 minutes after the start of each meal and measure their plasma acetaminophen concentration at 0, 15, 30, 45, 60, 90, 120, 150, 180, 300, and 420 min after liquid acetaminophen consumption for the last 8 women.
- Subjects will be asked to rate their hunger, fullness, desire and capacity to eat on a 10-cm visual analog scale every hour during waking hours.
- Exercise will take place between 7 and 9 h and 14 and 16 h during the before-meals trial and between 11 and 13 h and 18 and 20 h during the after-meals trial.

- Blood for hormone measurements and glucose determinations with Beckman spectrophotometer will be collected at 30 minute intervals between 6h and 21h and at hourly intervals through the night except for times when the two main meals are offered and at the start and termination of exercise, when additional samples will be collected at 15 minute intervals. Blood will be placed into EDTA-coated test tubes containing Aprotinin (250 KIU/50 μl plasma) for later hormone and metabolite determinations. A total of 188 ml of blood with 1 heavy isotope glucose infusion will be collected in each trial. Total blood collection per subject in the two trials will be 376 ml with 1 heavy isotope infusion.

Table 1: List of analytical procedures for measuring metabolites and hormones

	Metabolite/ hormone	Measurement method	Kit manufacturer
Metabolites	glucose	Colorimetric assay	Thermo DMA
	Beta-OH-butyrate		Randox, GB
Glucoregulatory hormones	insulin	RIA	Linco Research Inc
	glucagon		

Measurement of hepatic glucose production (Isotope infusion and sample preparation)
For 17 women, heavy glucose will be infused two times during the each of the four trials, between 20:00 and 06:00 h on days 1 and 2. For one woman, the heavy glucose isotope will be infused between 20:00 and 06:00 h on day 2. For the last 13 women, the heavy glucose isotope will be infused between 3:00 h and 10:00 h on day 3. Three blood samples will be withdrawn right before the start of isotope infusion for the determination of background isotope enrichment from one of the catheters. This will next be followed by a primed constant rate of infusion of [6,6,-$^2H_2$] glucose (0.41 μmol/kg*min after a prime of 35 μmol/kg, Cambridge Isotope Laboratory) with a calibrated syringe pumps (Harvard Apparatus, South Natick, MA). Three blood samples will be drawn right before the infusion start (between 19:50 h and 20:00 h for the first 18 women and between 2:50 h and 3:00 h for the rest 13 women), at the end of equilibration period, (between 23:50 h and 0:00 h for the first 18 women and between 5:50 h and 6:00 h for the rest 13 women), and at the end of each heavy glucose infusion. Hourly blood samples in duplicate will be drawn between 0:55 and 0:60 h. Plasma samples (1 ml) will be deproteinized by adding 1 ml of $Ba(OH)_2$ and 1 ml of 0.3 N $Zn(SO)_4$. Tubes will be vortexed and incubated in ice bath for 20 min, after centrifugation at 4°C, 3,000 rpm for 15 min, the 0.5 ml of supernatant is placed in separate tubes for glucose determination and the water is removed by vacuum centrifugation. The aldonitrile acetate derivative of glucose is prepared by adding 100 μl of hydroxylamine hydrochloride solution (20 mg/ml in pyridine) to the dried sample. Acetic anhydride (Supelco, Bellefonte, PA) is added (75 μl) after a 30-min incubation at 100° C and incubation continues for another hour. Samples are then evaporated under nitrogen. Before injection into gas chromatograph-mass spectrometer, the samples are reconstituted with ethyl acetate.

Calculations
Since plasma glucose is not in steady state during exercise and after meals, the rate of glucose appearance (Ra_G) is a measure of hepatic glucose production. The Ra_G and the rate of glucose disappearance (Rd_G) will be calculated with the non-steady-state equation of Steele (27) modified for use with stable isotopes, as follows:

$$Ra_G= F-Vd[(C/(1+E)(dE/dt)]/E \quad Rd_G= Ra_G - Vd[dC/dt)(1+E) - C(dE/dt)]/ (1 + E)^2$$

where F is isotope infusion rate, Vd is volume of glucose distribution estimated to be 100 ml/kg , C is plasma concentration of the tracee, E is tracer isotopic enrichment, and dE/dt and dC/dt are the maximum rates of change of enrichment and concentration, respectively, with respect to time.

Statistical analyses
The number of subjects for each treatment condition needed to find statistical differences in the major endpoints of this study (i.e. hormone and metabolite concentrations) was calculated based on mean and standard deviation values for these variables obtained in our preliminary study (Table 2). Power calculations were done for the data collected in the preliminary study by using periods of interest when changes due to treatments were taking place. These periods were periprandial changes for insulin and ketones, postprandial period for insulin, and postabsorptive periods for plasma glucose. Power analyses suggest that the studies will be adequately powered with 10 subjects. Our experimental design provides 10 subjects for each trial condition (Table 1).

Table 2. Power calculation for both proposed studies

Δ in dependent variable during exercise relative to meals	Power	α	SD (% of M %Δ)	Observed Δ	# of subjects per condition
Plasma insulin	0.8	0.05	56.3	35.7 %	8
Plasma glucose	0.8	0.05	6.6	20%	4
Plasma ketones	0.8	0.05	0.04	256.2 %	1

A mixed-model ANOVA for repeated measures (time *treatment) will be used with SAS 9.1 software. This analysis was previously performed for the preliminary study and for data in two other clinical studies that produced a large number of sequential data points. Tukey's post hoc analysis will be used to determine differences between trials. P <0.05 will indicate significance.

E. PROTECTION OF HUMAN SUBJECTS

1. Population**:**

A total of 31 healthy postmenopausal women will be recruited to participate in the two studies. All subjects will be screened with a detailed health history and physical examination, routine blood tests including fasting plasma glucose and insulin, resting metabolic rate (RMR) will be obtained with V-Max respiratory gas apparatus (Viasys, Cardinal Health, Palm Springs, CA), body weight, height, and composition analysis by dual energy X-ray absorptiometry (Lunar DPX DEXA scanner, during their first study trial), and a TSH measurement for normal thyroid function.

The inclusion criteria for both studies will be:

- Postmenopausal status
- Age 50 to 65 years
- BMI between 20 and 30 kg/m^2.
- Good health status (normotensive, fasting glucose < 100 mg/dl, fasting insulin <5 mIU/ml, hematocrit > 32%, hemoglobin >12 mg/dl)
- Absence of restricted food intake
- Absence of endocrine and metabolic disorders requiring medication other than hormonally corrected hypothyroidism
- Absence of musculoskeletal disabilities that would prevent walking

The exclusion criteria for both studies will be:

- Presence of endocrine and metabolic disease requiring medication, other than hormonally corrected hypothyroidism
- prescription medications for endocrine or metabolic disease other than for hypothyroidism
- Presence of musculoskeletal disabilities that would prevent walking
- Smoking

- Active dieting
- Absence of listed inclusion criteria
- Unwillingness to follow study protocol.

2. Source of Research Materials:

Ratings of hunger and satiety will be collected with visual analog scales.
Blood samples will be collected and used for hormone and metabolite determinations.
Metabolism will be measured with indirect calorimetry
Body fat measurements will be made with DXA scans.

3. Recruitment / Informed Consent:

A comprehensive informed consent will be used.

4. Potential Risks:

The risks of participating in these two studies are minimal. They may include:

- Risks of joint injuries during walking
- Risks of chest paints in individuals with undiagnosed CHD
- Pain, bruising, or inflammation at the site of indwelling catheter
- Infection due to inadvertent contamination of infused nutrients
- Radiation exposure during DEXA scan
- Risks of acetaminophen (Tylenol):
  - o Skin rash or itching (5%)
  - o Constipation (5%)
  - o Nausea/Vomiting (10-20%)
  - o Headache (1-10%)
  - o Allergic reaction (hives, shortness of breath)
  - o Hepatotoxicity (liver failure). Taking more than 4 grams (= 4000mg) of acetaminophen daily and drinking more than 3 alcoholic beverages per day increases the risk for damage to your liver when you take acetaminophen.

5. Protection from Risks:

- Risks of joint injuries will be prevented or minimized by inclusion of a warm up and cool down, recommendations to wear appropriate walking shoes
- Physical Activity Readiness Questionnaire will help us identify conditions that could suggest risk of CHD. Such individuals will not be accepted into the study.
- Subjects will receive clearance from their physicians attesting that no cardiac pathology or physical condition exists that would make exercise potentially hazardous to their health. Healthy people should experience no cardiac distress with moderate intensity walking in this study. The preliminary fitness test will be carried out in the Michigan Clinical Research Unit within the University of Michigan Hospital and emergency medical care.
- Intravenous catheter insertion will be performed by skilled personnel in the MCRU. Standard sterile techniques for vein puncture will be used and regular examination of i.v. catheter sites will be done.

Pain associated with vein puncture is usually mild and short-lived.

- The radiation exposure during DEXA scan is less than 1 mRem. This is the amount of radiation exposure obtained during one day in the United States.
- Be sure to tell the study team if you take acetaminophen (Tylenol) outside of this study, including acetaminophen found in your prescription and over the counter products and if you drink more than 2-3 alcoholic beverages per day. Also, let the study team know if you have had an allergic reaction to acetaminophen in the past

6. Potential Benefits to the Subject or Others, and Risk in Relation to These Benefits:

This study has the potential to further the understanding of mechanisms through which exercise and diet facilitate reduction in plasma glucose. The study will provide useful behavioral strategies for lowering of basal plasma glucose in subjects at risk of type 2 diabetes. The risk to benefit ratio is excellent.

7. Importance of the Knowledge to be Gained and
Why Risks to the Subject are Reasonable in Relation to this Knowledge:

The findings will contribute to the fundamental body of knowledge on the effects of exercise on the mechanism of glucoregulation. In addition, the information gained may eventually be of value in developing recommendations for optimal strategies of using exercise to prevent risks of type 2 diabetes in postmenopausal women. Therefore the small risks amply justify the knowledge gained.

8. Test article (investigational new drug, device, or biologic):

Heavy glucose ([6,6,-$^2H_2$] glucose) sold by Isotec (Miamisburg, OH, a subsidiary of Sigma Chemical) will be infused intravenously. Heavy glucose is a non-radioactive, naturally occurring isotope of glucose that does not present any health risks. Acetaminophen (Tylenol) will be applied with each meal to measure the rate of gastric emptying. Acetominophen will be administered in liquid form (concentration 160 mg/5 ml) in the amount of 20 ml (640 mg acetaminophen) with each of two meals. Maximum safe daily dose of acetaminophen is 4000 mg as specified by manufacturer. The proposed dose in this study is 32% of the maximal safe daily dose.

9 INCLUSION OF WOMEN AND MINORITIES

1. A description of the subject selection criteria and rationale for selection in terms of the scientific objectives and proposed study design.

This study was designed to examine the physiological reasons for glucose-lowering effect of particular timing of exercise and meals in postmenopausal women because of prevalence of overweight/obesity and diabetes in this segment of the population.

2. A compelling rationale for proposed exclusion of any sex/gender.

Because of the increased risk of diabetes in overweight postmenopausal women, and a greater prevalence overweight/obesity and hypoactivity in this segment of population, this study focuses on postmenopausal women.

3. A description of proposed outreach programs for recruiting women in clinical research as participants.

Women will be recruited from the community through advertisements in the university and Ann Arbor newspapers.

4. Recruitment of minorities.

Minority subjects will be actively recruited from the community through advertisements in the university and Ann Arbor newspapers. We will seek to achieve minority representation that is commensurate to demographics of Ann Arbor and its immediate vicinity.

5. A description of proposed outreach programs for recruiting minorities in clinical research as participants.

To date I have been able to attract minority women to my studies by simply placing advertisements in the newspapers. In the pilot study, summarized in section 4, two out of nine subjects were African American, a proportion that exceeds local demographics. Having past study participants from ethnic minorities helps recruitment of additional subjects through word of mouth.

Tables for Reporting Race and Ethnicity Data

Study Title: Liver glycogen in control of plasma glucose and hunger

Total Planned Enrollment: 20

TARGETED/PLANNED ENROLLMENT: Number of Subjects			
Ethnic Category	Sex/Gender		
	Females	Males	Total
Hispanic or Latino	0	0	0
Not Hispanic or Latino	31	0	31
Ethnic Category Total of All Subjects*	31	0	31
Racial Categories			
American Indian/Alaska Native	0	0	0
Asian	0	0	0
Native Hawaiian or Other Pacific Islander	0	0	0
Black or African American	5	0	5
White	26	0	26
Racial Categories: Total of All Subjects *	31	0	31

6. The proposed dates of enrollment including timeframe and dates.

The two studies entail 40 trials. We anticipate to be able to perform 6 to 8 experiments per month which would allow us to complete blood and data collection within 8 to 9 months. Sample and data analyses will be performed concurrently with subject recruitment and data collection, but the number and volume of assays that will have to be performed will result in peak involvement in hormone assays during months 8 through 16 of this award. Data reduction and interpretation will largely occur during the final six months of the award.

Projected timetable for completion of the two studies

Period of the study	Project activities
1-2 months	Staff orientation and organization<br>Start subject recruitment<br>Start experiments at a rate of 6 to 8/month<br>Start sample analyses
2-12 months	Continue subject recruitment<br>Continue experiments at the rate of 8/month<br>Continue sample analyses
10-12 months	Data reduction and interpretation<br>Preparation of manuscripts and new grant applications<br>Final report
16-18 months	

INCLUSION OF CHILDREN (UNDER 21 YEARS) & JUSTIFICATION

This study was designed to examine hormonal, metabolic, and behavioral effects of exercise in postmenopausal women because of prevalence of obesity and diabetes in this segment of the population. that can be ameliorated with regular exercise. Because children and adolescents have lower incidence of obesity and type 2 diabetes and very different endocrine physiology, they are not included in this study.

F. DATA SAFETY MONITORING PLAN.

Introduction

This research study, using 26 randomly assigned subjects, aims to test the hypotheses that (1) exercise before meals results in inadequate liver glycogen recovery, as manifested by increased ketone body production, due to increased exercise and post-exercise muscle glucose uptake and (2) that muscle glucose uptake following two spaced one-hour exercise bouts combined with a 50% lower carbohydrate content of the diet, will be of sufficient magnitude to produce inadequate hepatic glycogen repletion, reduced hepatic glucose production, and sustained lowering of blood glucose. The intervention in this study includes intravenous infusion of the naturally occurring heavy isotope of glucose. This protocol is deemed to pose low risk to participants. Because of this low-risk status, the data safety monitoring (DSM) plan for this trial focuses on close monitoring by the principal investigator (PI) and her research team that includes a physician, Dr. Charles Burant, along with prompt reporting of serious adverse events (SAEs), un-anticipated adverse events (AEs) as well as of other reportable incidents or occurrences (ORIOs) to the IRB-MED and General Clinical Research Center (GCRC) at the University of Michigan and to the NIDDK.

This DSM plan provides:
(1) Qualifications and responsibilities of the safety officer and of the team physician;
(2) Confidentiality and data storage;
(3) Review and reporting of study parameters;
(4) Monitoring and reporting of SAEs, AE, and ORIOs;
(5) Stopping rules.

(1) Qualifications and responsibilities of the Team Physician

The physician on the research team is Charles Burant, M.D., an Associate Professor in Department of Internal Medicine at the University of Michigan Medical School. Dr. Burant's specialty is Endocrinology and Metabolism, and he is directly involved in design and execution of the study and in evaluation of subjects and study results.

The experimental and health outcomes will be evaluated by Dr. Burant and members of the research team at monthly intervals to determine whether there is a need for any corrective action, ad hoc review, stopping of the study, or reports of specific out-of-range laboratory data to the University of Michigan IRB-MED or the MCRU.

(2) Confidentiality and data storage

Research data in the proposed study will be collected, stored, protected and analyzed in the following way. All demographic and health data from the PARQ and Physician's Release forms are filed in folders under subjects' names. These are securely stored under lock and key in file cabinets and in computer drives that are accessible only to the PI. PI distributes appropriately coded sets of blood samples for measurements, and data for calculations. All members of the study team have completed mandatory training in the protection of human research participants per guidelines issued by the U.S. Department of Health and Human Services, Office for Human Research Protections.

(3) Review and reporting of study parameters

Subject recruitment, compliance to the target demographic profile of subjects, adherence to protocol, and incidence of adverse events and other reportable incidents and occurrences will be reviewed monthly.

The monthly reviews will be conducted in a semi-formal manner with the date, time and place determined on a routine scheduled basis. The study team (PI, team physician, and study coordinator) will review incidence of ORIOs, AEs, the rate of subject accrual, and the rate and reasons for subject dropouts; adherence to protocol regarding target demographics, overall adherence to the study protocol, and any out-of-range data. The outcome of the monthly reviews will be implementation of corrective measures, if needed, to reduce AEs, dropouts, ORIOs and to improve accrual. Although part of the monthly process, e PI will do an additional review of annual data for completeness and accuracy, as well as protocol compliance. The results of this annual review will be sent to the University of Michigan IRB-MED and GCRC and to the NIDDK as part of annual continuation request.

(4) Monitoring and reporting of SAEs, AEs, and ORIOs;

All AEs, including SAEs, will be evaluated by the research team (PI, team physician and study coordinator) immediately upon their occurrence. PI and/or team physician will discuss SAEs within 24 h, and AEs within 72 h, of their occurrence. The PI/MD will determine relatedness of the AE with the protocol procedure (as definitely, probably, or possibly related, unlikely to be related or definitely not related).

We will use the definitions of adverse events (AEs) and other reportable incidents or occurrences (ORIOs) mandated by the University of Michigan IRB-MED (attached and also accessible at http://www.med.umich.edu/irbmed/ae_orio/ae_report_standard.htm)):

Adverse Events (AEs): are events that involve physiological, social, or psychological harm to subjects or risks of harm to additional subjects or others. AEs include expected and unexpected harmful effects, and unexpected risks of an interaction or an intervention.

Serious Adverse Event (SAE) is any untoward occurrence that: results in death, is life-threatening requires inpatient hospitalization or prolongation of existing hospitalization, results in persistent or significant disability/incapacity, or is a congenital anomaly/birth defect. (ICH Guidelines)

We will use the following AE Severity Grading Scale for Adverse Events:
Mild: Noticeable to the subject, does not interfere with the subject's daily activities, usually does not require additional therapy, dose reduction, or discontinuation of the study.
Moderate: Interferes with the subject's daily activities, possibly requires additional therapy, but does not require discontinuation of the study.
Severe: Severely limits the subject's daily activities and may require discontinuation of the study. This would include all adverse events defined as "Serious" by the IRBMED.

We will apply the above classification of AEs to our protocols as shown in Table 2:

Table 2: Classification of possible AEs and ORIOs in the proposed studies

**ORIOs**	**Mild AEs**	**Moderate AEs**	**Severe AEs**
**Protocol deviations**	**Muscle soreness**	**Joint sprain requiring medical intervention and healing**	**Injury requiring long-term intervention**
**Accidents or incidents**	**Dizziness during exercise requiring no medical intervention**	**Faintness requiring intervention**	**Cardiac distress requiring urgent intervention**
**Complaints**	**Subcutaneous infiltration of normal saline infusion**	**Subcutaneous infiltration of parenteral nutrient infusion fluids**	**Embolism from parenteral infusion**

We anticipate low incidence of adverse events as heavy glucose is a naturally occurring isotope and is metabolized in the same way as normal atomic weight hlucose. Subjects with overt cardiovascular or metabolic risk factors are excluded on the basis of preliminary health screens described in the grant proposal.

Reporting of SAEs, AEs, unexpected side effects, and ORIOs
We will follow the AE/ORIO reporting rules as defined by the University of Michigan IRB-MED (see the attached document AE/ORIO: Standard AE Reporting) that were modified to also meet NIDDK reporting requirements (see the attachment and table 3 below.)

Table 3: Reporting of AEs, SAEs or ORIOs to IRB-MED, GCRC, and NIDDK

**Event type**	**Frequency of reports to SO**	**Frequency of reports to UM IRB-MED & GCRC), NIDDK**
Serious or severe adverse events	Discussion with team physician within 24 hours	Report within 7 days of the event or notification of the event to UM
Moderate AE possibly, probably or related<br>Moderate AE unlikely or unrelated	Discussion with team physician within 72 hours	Report within 7 days of the event or notification of the event to UM<br>Report with annual report

Mild AEs regardless of relatedness	Notification at the time of monthly meeting	Report with annual report
ORIOs-urgent subject safety or regulatory concern	Discussion with team physician within 72 hours	Report within 7 days of the event or notification of the event to UM
ORIOs-situation/event potentially alters risk-benefit assessment or the integrity of the study	Discussion with team physician within 72 hours	Report within 7 days of the event or notification of the event to UM
ORIOs- all other	Discussion with team physician at the time of the monthly meeting	Report with annual report

(5) Stopping rules

Stopping rules will focus on the unlikely event of severe cardiac distress The study team, will evaluate and submit recommendation for stopping or continuing the study to the IRBMED and MCRU. The IRBMED will be the decision making committee. MCRU input will be submitted to the IRBMED.

G. REFERENCES CITED

1. Schernthaner G. Matthews DR. Charbonnel B. Hanefeld M. Brunetti P. Quartet [corrected] Study Group. Efficacy and safety of pioglitazone versus metformin in patients with type 2 diabetes mellitus: a double-blind, randomized trial. J Clin Endocr Metab. 89(12):6068-76, 2004 Dec.
2. Knowler WC, Barrett-Connor NF, Fowler SE, Hamman RF, Lachin JM, Walker EA, Nathan D (2002) Reduction in the incidence of type 2 diabetes with lifestyle intervention or metformin. New Engl J Med 346: 393-403.
3. Richter EA, Ploug T, Galbo H (1985) Increased muscle glucose uptake after exercise. No need for insulin during exercise. Diabetes 34:1041-1048.
4. Ahlborg G, Felig P, Hagenfeldt L, Hendler R, Wahren J. Substrate turnover during prolonged exercise in man. J Clin Invest 53: 1080-1090, 1974.
5. Ahlborg G, Felig P. Lactate and glucose exchange across the forearm, legs, and splanchnic bed during and after prolonged leg exercise. J Clin Invest 69: 45-54, 1982.
6. Coyle EF, Coggan AR, Hemmert MK, Ivy JL. Muscle glycogen utilization during prolonged strenuous exercise when fed carbohydrate, J Appl Physiol 61: 165-172, 1986.
7. Galbo H, Christensen NJ, Holst JJ. Glucose-induced decrease in glucagon and epinephrine responses to exercise in man. J Appl Physiol 42: 525-530, 1977.
8. Galbo H. Holst JJ. Christensen NJ (1977) The effect of different diets and of insulin on the hormonal response to prolonged exercise**.** Acta Physiol Scand 107:19-32.
9. Sandoval DA, Davis SN. Metabolic consequences of exercise-associated autonomic failure. Exerc Sport Sci Rev 34: 72-76, 2006.
10. Galassetti P, Mann S, Tate D, Neil RA, Wasserman DH, Davis, SN. Effect of morning exercise on counterregulatory responses to subsequent, afternoon exercise. J Appl Physiol 91:91-99, 2001.
11. Sandoval D, Aftab-Guy D, Richardson A, Erd AC, Davis SN (2004) Effects of same day exercise on subsequent counterregulatory responses to hypoglycemia. Diabetes 53:A88.
12. Galassetti P, Mann S, Tate D, Neill RA, Costa F, Wasserman DH, Davis SN. Effects of antecedent prolonged exercise on subsequent counterregulatory responses to hypoglycemia. Am J Physiol 280: E908-E917, 2001.

13. Sandoval DA, Aftab-Guy D, Richardson MA, Davis SN (2004) Effects of low and moderate antecedent exercise on counterregulatory responses to subsequent hypoglycemia in type 1 diabetes. Diabetes 53: 1798-1806.
14. Sandoval DA, Aftab-Guy D, Richardson MA, Ertl AC, Davis SN (2006) Acute same-day effects of antecedent exercise on counterregulatory response to subsequent hypoglycemia in type 1 diabetes mellitus. Amer J Physiol 290: E1331-E1338.
15. Galbo H. Christensen NJ. Mikines KJ. Sonne B. Hilsted J. Hagen C. Fahrenkrug J (1981) The effect of fasting on the hormonal response to graded exercise. J Clin Endocr Metab 52:1106-1112.
16. Kaciuba Uscilko H, Kruk B, Szczypaczewska M, Opaszonski B, Stupnicka E, Bicz B, Nazar K. Metabolic, body temperature and hormonal responses to repeated periods of prolonged cycle ergometer exercise in man. Eur J Appl Physiol 64: 26-31, 1992.
17 Saltin B, Wahren J, Pernow (1974) Phosphagen and carbohydrate metabolism during exercise in trained middle aged men. Scand J Clin Invest 33: 71-77.
18. Romijn JA, Coyle EF, Sidossis LS, Gastaldelli A, Horowitz JF, Endert E, Wolfe RR (1993) Regulation of endogenous fat and carbohydrate metabolism in relation to exercise intensity and duration. Am J Physiol 265:E380-E391.
19. Goodyear LJ, Kahn BB (1998) Exercise, glucose transport, and insulin sensitivity. Annu Rev Med 49: 235-261.
20. Wojtaszewski JF, Hansen BF, Gade, Kiens B, Markuns JF, Goodyear LJ, Richter EA (2000) Insulin signaling and insulin sensitivity after exercise in human skeletal muscle. Diabetes 49: 325-331.
21. Jensen J, Jebens E, Brennesvik EO, Ruzzin J, Soos MA, Engebretsen EM, O'Rahilly S, Whitehead JP (2006) Muscle glycogen inharmoniously regulates glycogen synthase activity, glucose uptake, and proximal insulin signaling. Am J Physiol 290:E154-E162.
22. Newsholme EA, Leech ER (1983) Biochemistry for the medical sciences, New York: John Wiley & Sons, pp 178-185.
23. Fell RD. McLane JA. Winder WW. Holloszy JO. Preferential resynthesis of muscle glycogen in fasting rats after exhausting exercise. Amer J Physiol 238:R328-32, 1980.
24. Bergström J, Hultman E (1966) Muscle glycogen synthesis after exercise: An enhancing factor localized to the muscle cells in man. Nature 210: 309-310.
25. Borer KT, Wuorinen E, Burant C, Lukos J, Denver J, Porges SW. Two bouts of exercise before meals, but not after meals, lower daily blood glucose in nondiabetic women. Diabetologia (under review).
26. Adams JH, Koeslag JH (1988) Carbohydrate homeostasis and post-exercise ketosis in trained and untrained rats. J Physiol 407: 453-461.
27. Koeslag JH (1982) Post-exercise ketosis and the hormone response to exercise: a review. Med Sci Sports Exer 14: 327-334.
28. Koeslag JH, Noakes TD, Sloan AW (1982) The effects of alanine, glucose and starch ingestion on the ketosis produced by exercise and starvation. J Physiol 325: 363-376.
29. Koeslag JH. Noakes TD, Sloan AW (1980) Post-exercise ketosis. J Physiol 301: 79-90.
30. Heading RC, Nimmo J, Prescott LF, Tothill P. The dependence of paracetamol absorption on the rate of gastric emptying. Br J Pharmacol. 1973;47:415-21.

Research Protocol for The exercise effects on Appetite and gut peptides while on high-fat diet (HUM00043233)

ABSTRACT

Two spaced bouts of moderate-intensity exercise reduce hunger and increase secretion of GIP (glucose-dependent insulinotropic hormone) and GLP-1 (glucagon-like peptide-1) after the morning meal but not after the afternoon meal to a greater extent in subjects eating 30% carbohydrate and 45% fat diet compared to subjects eating 60% carbohydrate and 25% fat diet (Lin et al., 2010, unpublished data). To determine whether the effects are due to macronutrient difference or the combination of diet and concurrent exercise, we need to compare the exercise high-fat trial to a sedentary high-fat trial. The sedentary dietary control was not done in the original study (protocol HUM00017875).

Research Objectives:

Determine whether the mid-day suppression of hunger and amplified increase in the release of GIP and GLP-1 following morning exercise is due to increased fat content of the diet per se or a combination of high fat diet after morning exercise. The action of gut peptides, particularly GLP-1, on gastric emptying is likely to be important in mediating its effects on postprandial appetite and glycemia (Nauck et al. 1997). Our hypothesis is that exercise amplifies gut peptide secretion when diet is enriched with fat, and that this stimulus suppresses the hunger sensation.

PROPOSAL NARRATIVE

A. SPECIFIC AIMS

Specific aim: Determine whether a change in macronutrient composition from 60% carbohydrate and 25% fat to 30% carbohydrate and 45% fat is responsible by itself for suppression of hunger and increased secretory response of GIP and GLP-1, or whether these changes depend on preceding exercise. We will measure (a) concentrations of plasma GIP (glucose-dependent insulinotropic hormone) and GLP-1 (glucagon-like peptide-1) by chemiluminescent multiplex assay, (b) concentrations of plasma ancetaminophen to assess the gastric emptying rate, (c) concentrations of plasma insulin, and glucagon by radioimmunoassay, and glucose, ketone bodies, and free fatty acids with appropriate spectrophotometric methods, (d) hourly appetite responses with visual analog scale under two conditions: sedentary (SED) and exercise (EX).

Hypothesis: Hunger suppression and secretion of GIP and GLP-1 after the morning meal will be greater with slower gastric emptying rate when a meal consisting of 45% fat and 30% carbohydrate follows three hours after a 2-hour bout of moderate-intensity exercise than in the absence of exercise.

B. BACKGROUND AND SIGNIFICANCE

There is currently no clear understanding of the contribution of gut peptides to the control of hunger (Blundell et al., 2008; Edholm et al., 2010), the effect of exercise on the secretion of gut peptides (Chanoine et al., 2008; Adam and Westerterp-Plantenga, 2004), and the possible interaction of the two variables in the control of human hunger. The lack of understanding of the mechanism of hunger is the core obstacle to curbing the recent epidemic of obesity (Flegal et al, 2009). Rapid rise in obesity in the US is to a large extent a consequence of a lifestyle of eating more calories as well as selecting high-energy and high-fat food. There is incomplete information to date how increased fat composition of the diet affects human appetite (Cecil et al., 1999; Johnson and Vickers, 1993). There is even less information regarding whether the high-fat food modifies the responses of human appetite to exercise (King et al., 2009). Exercise is associated with suppression of hunger, during (Borer et al 2005, 2009) and for about one hour following (King et al., 1994) a bout of exercise entailing expenditure of about 300 to 500 Kcal.

There currently is a great deal of interest in the possible control of hunger by gastrointestinal (GI) peptides in general, and GLP-1 and PYY, in particular. GI peptides in general mediate nutrient digestion, usually slow down nutrient transit, and contribute to satiation by acting on the vagus nerve through its projection to the nucleus of the solitary tract in the brainstem (Berthoud 2008). Intravenous infusion of GLP-1 at 0.75 pM/min markedly delayed gastric emptying, decreased plasma glucose and insulin levels, and markedly increased insulinogenic index. It also reduced appetite sensations (Edholm et al., 2010). GIP infusion at 5 pM/min in the same study, had no impact on appetite, accelerated gastric emptying, lowered plasma glucose and insulin, and increased insulinogenic index. How diet-and exercise-induced changes in the prandial response of these two peptides are currently not known. The contention that the GI peptide ghrelin mediates hunger and initiates feeding (Wren et al., 2001) has not been consistently supported by weight losses achieved by different means (Valderas et al., 2010). There is also an interest in the role of exercise on gut peptide secretion. King et al. (2009) found that exercise increased both overall drive to eat and the satiating efficiency of food. Exercise training could also stimulate the secretion of GLP-1(Adam and Westerterp-Plantenga, 2004) and might increase the magnitude of the postprandial GLP-1 response as well (Chanoine et al. 2008)

To help individuals reduce weight by effective use of exercise and diet, it is necessary to increase our understanding of how dietary composition per se or diets in conjunction with exercise influence human hunger.

C. PRELIMINARY STUDIES

In the course of pursuing a different specific aim in the protocol HUM00017875, we have also measured psychophysical manifestations of appetite on a visual analog scale and changes in plasma concentration of several gut hormones, including GIP, GLP-1, and PYY. In 24 trials with four experimental conditions: sedentary (SED), exercise before the meals (EBM), both with a low-fat, high-carbohydrate-diet (60% and 25%, respectively), and modified exercise (1 hour instead of 2 hours before the meals) along with modified diet (30% carbohydrate and 45% fat) (MOD condition), and exercise after the meals (EAM), we have discovered that there was a greater suppression of hunger (Figure 1) and greater increase in GLP-1 (Figure 2) and GIP (Figure 3) secretion after the morning meal in the higher fat condition. Modified diet resulted in the elevation of plasma PYY concentration at all measurement times unrelated to the treatments (data not shown).

Figure 1. Hunger

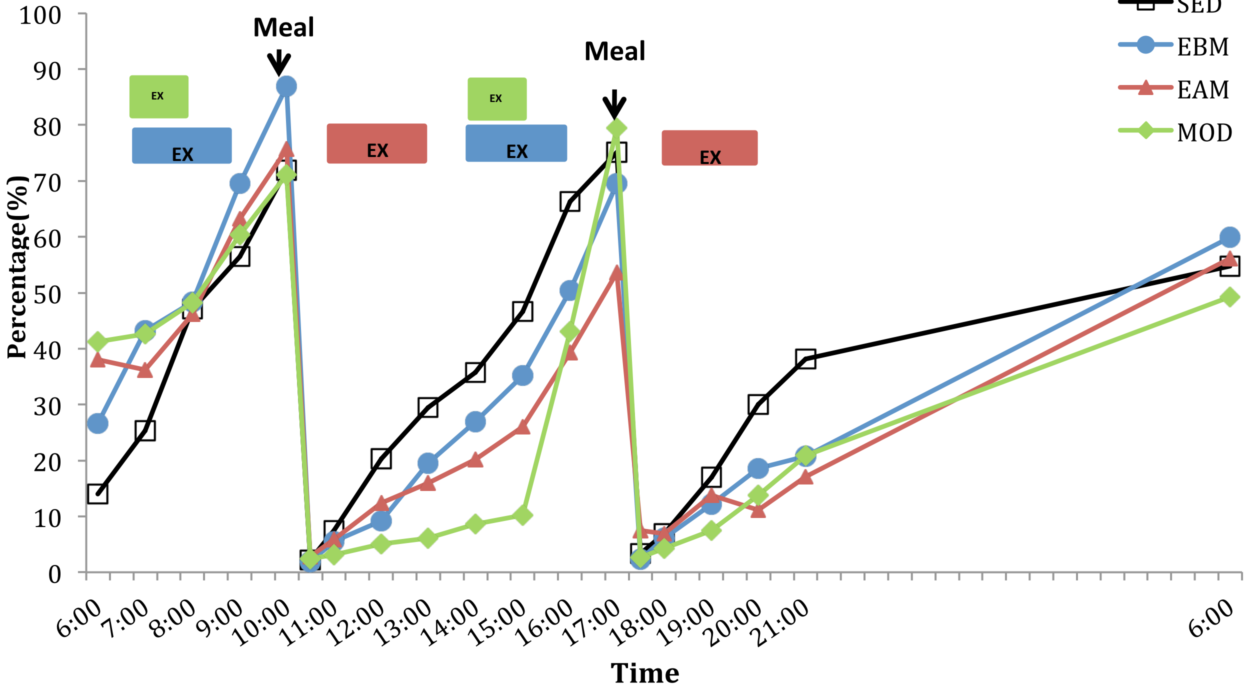

Figure 2. Plasma GLP-1

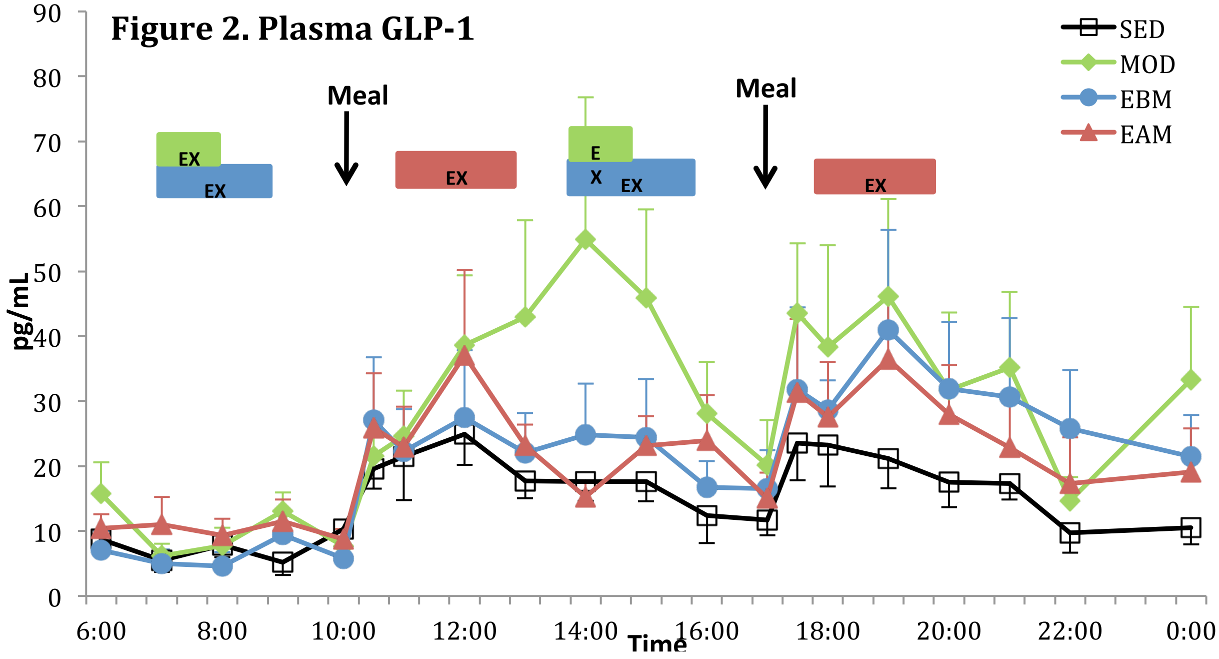

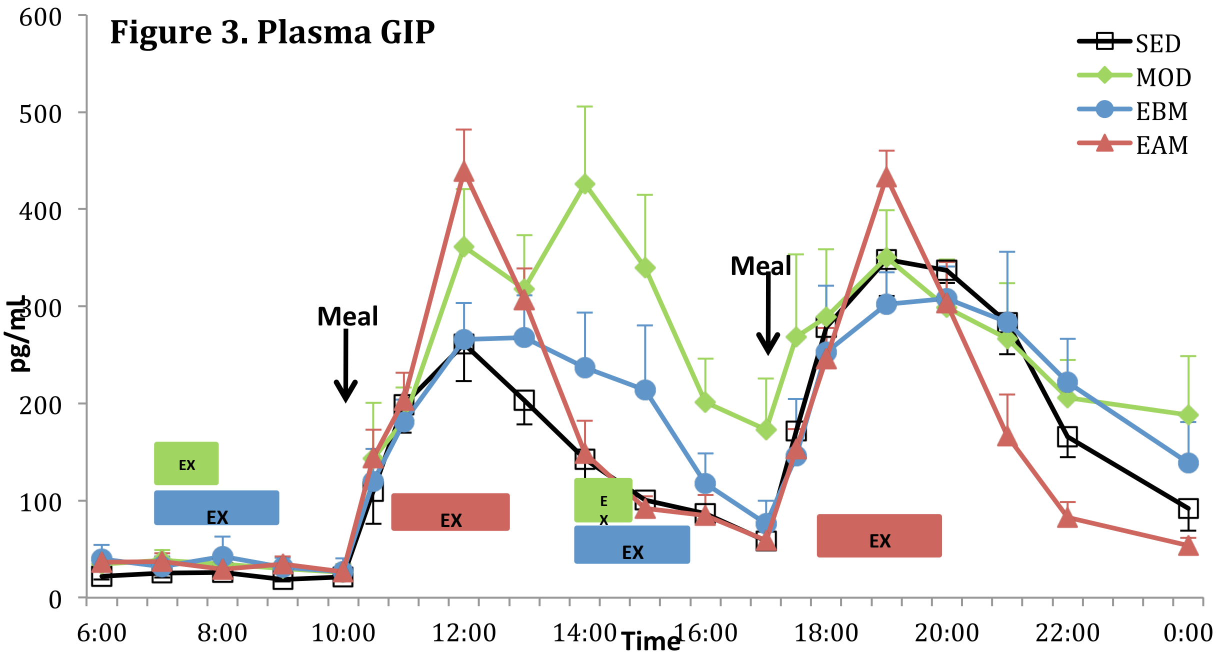

The present study adds two necessary additional treatments to allow for a comparison of the relative contribution of diet composition alone, or in combination with exercise, possible by contrasting the results in the protocol HUM00017875 performed with the high carbohydrate (60%), low fat (25%) diet to the present protocol with low carbohydrate (30%), high-fat (45%) diet. The proper comparison between the effects of diet and exercise in the two protocols will be possible by having subjects exercise for two hours with high-fat diet while in the protocol HUM00017875 exercise duration was only 1 hour. The sedentary high-fat diet treatment in the proposed study also is necessary to assess the possible hunger suppressive and gut peptide stimulatory effect of high-fat diet alone. This condition was not present in protocol HUM00017875 where sedentary subjects were given high-carbohydrate, low-fat diet. Thus, two-hours of exercise and sedentary condition with high-fat diet treatment in the proposed study are necessary to assess whether the effect on hunger and gut peptide secretion are contingent of precedent exercise of the same duration as in the protocol HUM00017875 or on change in diet composition alone.

D. <u>RESEARCH DESIGN AND METHODS</u>

Subjects

14 postmenopausal non-diabetic women of similar characteristics as in original study will be recruited. All subjects will be screened with a detailed history and physical examination, routine blood tests including CBC to exclude anemia and TSH to exclude hypothyroidisms, and body weight, height, and body composition analysis by dual-energy x-ray absorptiometry (DEXA). Subject fasting plasma glucose will be less than 100 mg/dl. The exclusion criteria will be metabolic disease and prescription medications other than for hypothyroidism. All subjects will sign an informed consent document approved by the University of Michigan Medical School Institutional Review Board.

Figure 4: General study design

- Studies will be performed in the Michigan Clinical Research Unit (MCRU) where subjects will be admitted at 18 h before the study day and receive a high-fat evening meal at 19 h containing 30% carbohydrate, 25% protein, and 45% fat. The energy content of the meal will be 30 Kcal/kg ideal body weight and total calories will be one third of daily amount. Each hospital stay during an individual trial will last 36 h

- To match the trial assignment done in the protocol HUM00017875, to which the proposed study data will be compared, we will randomly assign two subject to two trials (both in exercise and sedentary) and 12 more subjects will be assigned to single trials, six to EX, and six to SED. This will result in 8 exercise and 8 sedentary data sets that are needed for achieving statistical power.
- Two catheters will be inserted into contralateral arm veins at 19:30 h before the study day, one for blood collection and the other for glucose of a small amount (approximate 4 grams per each 10-hour nocturnal period) of regular glucose to match the amounts and infusion times of stable glucose isotope in the study HUM00017875.
- Peak aerobic capacity of the subject will be determined before the start of the study from a screening treadmill test. This test involves collection of ventilatory and respirometric measurements from the subject during progressive increases in treadmill slope (2% every 3 minutes) while walking on a level treadmill at 3 mph. During this screening test, as well as during the exercise portion of the study, the subject will breathe through a mouthpiece equipped with a two-way valve while the rate of ventilation, oxygen consumption (VO_2) and carbon dioxide production (VCO_2) are measured by indirect calorimetry. The results of this test will allow us to determine the walking speed at 45 % of subject's peak effort.
- Energy expenditure of exercise will be measured for 30 min at hourly intervals from oxygen (O_2) and carbon dioxide (CO_2) concentration that are continuously sampled from a mixing-chamber and analyzed by the (AEI Technologies, Pittsburgh, PA). The VO_2 and the respiratory quotient (ratio of VCO_2 over VO_2) obtained allow the calculation of calories expended during exercise and the contribution of carbohydrates and lipids to the energy expenditure (Saltin et al., 1974; Koeslag et al., 1980).
- Resting metabolic rate (RMR) will be obtained with V-Max respiratory gas apparatus (Viasys, Cardinal Health, Palm Springs, CA) between 6 and 6:30 h and after both meals, 10:30-11 h and 17:30-18h, on the day of the experiment and between 6 and 6:30 h on the morning after the completion of the study. RMR is assessed with the help of a canopy placed over subject's head, allowing measurements of breathing rate and respiratory gas analysis.
- Meals will be provided at 10 and 17 h on study day. Macronutrient composition of meals will be 30% carbohydrate, 25% protein, and 45% fat. Energy content of the meals will be 25 Kcal/kg body weight and each meal will provide one half of the daily amount. We also provide 20ml of oral liquid acetaminophen (160mg/5ml), which has been used as a marker drug to study the rate of gastric emptying (Heading et al. 1973), 30 minutes after the start of each meal and measure their plasma acetaminophen concentration at 0, 15, 30, 45, 60, 90, 120, 150, 180, 300, and 420 min after liquid acetaminophen consumption.
- Subjects will be asked to rate their hunger, fullness, desire and capacity to eat on a 10-cm visual analog scale every hour during waking hours.
- Exercise will take place between 7 and 9 h and 14 and 16 h during the exercise trial (EX) and subject remained sedentary during control trial (SED).
- Blood for hormone measurements and glucose determinations with Beckman spectrophotometer will be collected at 30 minute intervals between 6h and 20h and at hourly intervals through the night except for times when the two main meals are offered and at the start and termination of exercise, when additional samples will be collected at 15 minute intervals. Blood will be placed into EDTA-coated test tubes containing Aprotinin (250 KIU/ ml blood) and dipeptidyl peptidase inhibitor IV (10 μl/ml blood) for later hormone and metabolite determinations. A total of 225 ml of blood will be collected in each trial.

Table 1: List of analytical procedures for measuring metabolites and hormones

	Metabolite/ hormone	Measurement method	Kit manufacturer
Metabolites	glucose	Colorimetric assay	Thermo DMA
	Beta-OH-butyrate		Randox, GB

Gut peptides,	GLP-1, GIP, PYY	Chemiluminsecent multiplex assay	Milolipore Corp (Linco Research)
Glucoregulatory hormones	insulin	RIA	Linco Research Inc

Statistical analyses
The number of subjects for each treatment condition needed to find statistical differences in the major endpoints of this study (i.e. hormone and metabolite concentrations) was calculated based on mean and standard deviation values for these variables obtained in our preliminary study (Table 2). Power calculations were done for the data collected in the preliminary study by using periods of interest when changes due to treatments were taking place. These periods were periprandial changes for insulin and ketones, postprandial period for insulin, and postabsorptive periods for plasma glucose. Power analyses suggest that the studies will be adequately powered with 8 subjects. Our experimental design provides 8 subjects for each trial condition (Table 1).

Table 2: Power calculation for both proposed studies

Δ in dependent variable during exercise relative to meals	Power	α	SD (% of M %Δ)	Observed Δ	# of subjects per condition
Plasma insulin	0.8	0.05	56.3	35.7 %	8
Plasma GLP-1	0.8	0.05	50	100%	7
Plasma GIP	0.8	0.05	43.1	86.2%	7
Plasma PYY	0.8	0.05	25.45	50.9%	6
Plasma glucose	0.8	0.05	6.6	20%	4
Plasma ketones	0.8	0.05	0.04	256.2 %	1

A mixed-model ANOVA for repeated measures (time *treatment) will be used with SAS 9.2 software. Tukey's post hoc analysis will be used to determine differences between trials. $P < 0.05$ will indicate significance.

E. PROTECTION OF HUMAN SUBJECTS

1. Population**:**

A total of 14 healthy postmenopausal women will be recruited to participate in one (n=2) or two (n=12) trials. All subjects will be screened with a detailed health history and physical examination, routine blood tests including fasting plasma glucose, CBC to exclude anemia, TSH to exclude hypothyroidism, and body weight, height, and composition analysis by dual energy X-ray absorptiometry (Lunar DPX DEXA scanner).

The inclusion criteria for both studies will be:

- Postmenopausal status
- Age 50 to 65 years
- BMI between 20 and 30 kg/m^2.
- Good health status (normotensive, fasting glucose < 100 mg/dl, , hematocrit > 32%, hemoglobin >12 mg/dl)
- Absence of restricted food intake
- Absence of endocrine and metabolic disorders requiring medication other than hormonally corrected hypothyroidism
- Absence of musculoskeletal disabilities that would prevent walking

The exclusion criteria for both studies will be:

- Presence of endocrine and metabolic disease requiring medication, other than hormonally corrected hypothyroidism
- Presence of musculoskeletal disabilities that would prevent walking
- Smoking
- Active dieting
- Absence of listed inclusion criteria
- Unwillingness to follow study protocol.

2. Source of Research Materials:

Ratings of hunger and satiety will be collected with visual analog scales.
Blood samples will be collected and used for hormone and metabolite determinations.
Metabolism will be measured with indirect calorimetry. Body fat measurements will be made with DEXA scans.

3. Recruitment / Informed Consent:

A comprehensive informed consent will be used.

4. Potential Risks:

The risks of participating in these two studies are minimal. They may include:

- Risks of joint injuries during walking
- Risks of chest pain in individuals with undiagnosed CHD
- Pain, bruising, or inflammation at the site of indwelling catheter
- Infection due to inadvertent contamination of infused nutrients
- Radiation exposure during DEXA scan

6. Protection from Risks:

- Risks of joint injuries will be prevented or minimized by inclusion of a warm-up and cool-down, and recommendations to wear appropriate walking shoes
- Physical Activity Readiness Questionnaire will help us identify conditions that could suggest risk of CHD. Such individuals will not be accepted into the study.
- Subjects will receive clearance from their physicians attesting that no cardiac pathology or physical condition exists that would make exercise potentially hazardous to their health. Healthy people should experience no cardiac distress with moderate intensity walking in this study. The preliminary fitness test will be carried out in the Michigan Clinical Research Unit within the University of Michigan Hospital and emergency medical care.
- Intravenous catheter insertion will be performed by skilled personnel in the MCRU. Standard sterile techniques for vein puncture will be used and regular examination of i.v. catheter sites will be done. Pain associated with vein puncture is usually mild and short-lived.
- The radiation exposure during DEXA scan is less than 1 mRem. This is the amount of radiation exposure obtained during one day in the United States.

6. Potential Benefits to the Subject or Others, and Risk in Relation to These Benefits:

This study has the potential to further help individuals reduce weight by effective use of exercise and diet as behavioral strategies to prevent obesity and other related chronic diseases. It is necessary to increase

our understanding of the role of dietary composition alone or diet in conjunction with exercise in the control of gut peptide secretion and human hunger.

7. Importance of the Knowledge to be Gained and Why Risks to the Subject are Reasonable in Relation to this Knowledge:

The findings will contribute to the fundamental body of knowledge on the effects of exercise on the mechanism of appetite control and gut peptides regulation. In addition, the information gained may eventually be of value in developing recommendations for optimal strategies of using exercise and diet to prevent risks of obesity and other related diseases in postmenopausal women. Therefore the small risks amply justify the knowledge gained.

8. Test Article (investigational new drug, device, or biologic): None

9 INCLUSION of WOMEN AND MINORITIES

1. A description of the subject selection criteria and rationale for selection in terms of the scientific objectives and proposed study design.

This study was designed to examine the mechanism through which diet composition and exercise control gut peptide secretion and appetite in postmenopausal women because of prevalence of overweight/obesity and diabetes in this segment of the population.

2. A compelling rationale for proposed exclusion of any sex/gender.

Because of the increased risk of diabetes in overweight postmenopausal women, and a greater prevalence overweight/obesity and hypoactivity in this segment of population, this study focuses on postmenopausal women.

3. A description of proposed outreach programs for recruiting women in clinical research as participants.

Women will be recruited from the community through advertisements posted on the UMClinicalstudies website and Ann Arbor newspapers.

4. Recruitment of minorities.

Minority subjects will be actively recruited from the community through advertisements in the university and Ann Arbor newspapers. We will seek to achieve minority representation that is commensurate to demographics of Ann Arbor and its vicinity.

5. A description of proposed outreach programs for recruiting minorities in clinical research as participants.

To date I have been able to attract minority women to my studies by simply placing advertisements in the newspapers. In one of my previous study (MCRU Protocol 1802), two out of nine subjects were African American, a proportion that exceeds local demographics. Having past study participants from ethnic minorities helps recruitment of additional subjects through word of mouth.

Table 3: Race and Ethnicity Data Report

Study Title: The Exercise Effects on Appetite and Gut Peptides while on High-Fat Diet

Total Planned Enrollment: 14

TARGETED/PLANNED ENROLLMENT: Number of Subjects			
Ethnic Category	Sex/Gender		
	Females	Males	Total
Hispanic or Latino	1	0	1
Not Hispanic or Latino	13	0	13
Ethnic Category Total of All Subjects*	14	0	14
Racial Categories			
American Indian/Alaska Native	0	0	0
Asian	0	0	0
Native Hawaiian or Other Pacific Islander	0	0	0
Black or African American	1	0	1
White	13	0	13
Racial Categories: Total of All Subjects *	14	0	14

6. The proposed dates of enrollment including timeframe and dates.
This study entails 16 trials. We anticipate to be able to perform 2 to 4 experiments per month which would allow us to complete blood and data collection within 9 months. Sample and data analyses will be performed concurrently with subject recruitment and data collection, but the number and volume of assays that will have to be performed will result in peak involvement in hormone assays during months 12 through 18 of this study. Data reduction and interpretation will largely occur during the final six months of the study.

Table 4: Projected timetable for completion of the two studies

Period of the study	Project activities
1-3 months	Staff orientation and organization<br>Start subject recruitment<br>Start experiments at a rate of 2 to 4/month<br>Start sample analyses
3-12 months	Continue subject recruitment<br>Continue experiments at the rate of 3/month<br>Continue sample analyses
12-18 months	Data reduction and interpretation<br>Preparation of manuscripts and new grant applications<br>Final report

INCLUSION OF CHILDREN (UNDER 21 YEARS) & JUSTIFICATION

This study was designed to examine hormonal, metabolic, and behavioral effects of exercise in postmenopausal women because of prevalence of obesity and diabetes in this segment of the population that can be ameliorated with regular exercise. Because children and adolescents have different endocrine physiology, they are not included in this study.

F. DATA SAFETY MONITORING PLAN.

Introduction

This research study, using 14 randomly assigned subjects, aims to test the hypotheses that hunger suppression and secretion of GIP and GLP-1 after the morning meal will be greater when a meal consisting of 45% fat and 30% carbohydrate follows three hours after a 2-hour bout of moderate-intensity exercise than in the absence of exercise.

The intervention in this study includes intravenous infusion of a small amount of regular glucose (3-5 g/10 nocturnal hours during two nights). This protocol is deemed to pose low risk to participants. Because of this low-risk status, the data safety monitoring (DSM) plan for this trial focuses on close monitoring by the principal investigator (PI) and her research team that includes a physician, Dr. Charles Burant, along with prompt reporting of serious adverse events (SAEs), un-anticipated adverse events (AEs) as well as of other reportable incidents or occurrences (ORIOs) to the IRB-MED and General Clinical Research Center (GCRC) at the University of Michigan and to the NIDDK.

This DSM plan provides:
(1) Qualifications and responsibilities of the research team and of the team physician;
(2) Confidentiality and data storage;
(3) Review and reporting of study parameters;
(4) Monitoring and reporting of SAEs, AE, and ORIOs;
(5) Stopping rules.

(1) Qualifications and responsibilities of the Team Physician

The physician on the research team is Charles Burant, M.D., a Professor in Department of Internal Medicine at the University of Michigan Medical School. Dr. Burant's specialty is Endocrinology and Metabolism, and he is involved in health evaluation of subjects and study results.

The experimental and health outcomes will be evaluated by Dr. Burant nand members of the research team at monthly intervals to determine whether there is a need for any corrective action, ad hoc review, stopping of the study, or reports of specific out-of-range laboratory data to the University of Michigan IRB-MED or the MCRU.

(2) Confidentiality and data storage

Research data in the proposed study will be collected, stored, protected and analyzed in the following way. All demographic and health data from the PARQ and Physician's Release forms are filed in folders under subjects' names. These are securely stored under lock and key in file cabinets and in computer drives that are accessible only to the PI. PI distributes appropriately coded sets of blood samples for measurements, and data for calculations. All members of the study team have completed mandatory training in the protection of human research participants per guidelines issued by the U.S. Department of Health and Human Services, Office for Human Research Protections.

(3) Review and reporting of study parameters

Subject recruitment, compliance to the target demographic profile of subjects, adherence to protocol, and incidence of adverse events and other reportable incidents and occurrences will be reviewed monthly.

The monthly reviews will be conducted in a semi-formal manner with the date, time and place determined on a routine scheduled basis. The study team (PI, team physician, and study coordinator) will review incidence of ORIOs, AEs, the rate of subject accrual, and the rate and reasons for subject dropouts; adherence to protocol regarding target demographics, overall adherence to the study protocol, and any out-of-range data. The outcome of the monthly reviews will be implementation of corrective measures, if needed, to reduce AEs, dropouts, ORIOs and to improve accrual. Although part of the monthly process, the PI will do an additional review of annual data for completeness and accuracy, as well as protocol compliance. The results of this annual review will be sent to the University of Michigan IRB-MED and MCRU and to the NIDDK as part of annual continuation request.

(4) Monitoring and reporting of SAEs, AEs, and ORIOs;
All AEs, including SAEs, will be evaluated by the research team (PI, team physician and study coordinator) immediately upon their occurrence. PI and/or team physician will discuss SAEs within 24 h, and AEs within 72 h, of their occurrence. The PI/MD will determine relatedness of the AE with the protocol procedure (as definitely, probably, or possibly related, unlikely to be related or definitely not related).
We will use the definitions of adverse events (AEs) and other reportable incidents or occurrences (ORIOs) mandated by the University of Michigan IRB-MED (attached and also accessible at http://www.med.umich.edu/irbmed/ae_orio/ae_report_standard.htm)):

Adverse Events (AEs): are events that involve physiological, social, or psychological harm to subjects or risks of harm to additional subjects or others. AEs include expected and unexpected harmful effects, and unexpected risks of an interaction or an intervention.

Serious Adverse Event (SAE) is any untoward occurrence that: results in death, is life-threatening requires inpatient hospitalization or prolongation of existing hospitalization, results in persistent or significant disability/incapacity, or is a congenital anomaly/birth defect. (ICH Guidelines)

We will use the following AE Severity Grading Scale for Adverse Events:
Mild: Noticeable to the subject, does not interfere with the subject's daily activities, usually does not require additional therapy, dose reduction, or discontinuation of the study.
Moderate: Interferes with the subject's daily activities, possibly requires additional therapy, but does not require discontinuation of the study.
Severe: Severely limits the subject's daily activities and may require discontinuation of the study. This would include all adverse events defined as "Serious" by the IRBMED.
We will apply the above classification of AEs to our protocols as shown in Table 5:
Table 5: Classification of possible AEs and ORIOs in the proposed studies

ORIOs	Mild AEs	Moderate AEs	Severe AEs
**Protocol deviations**	**Muscle soreness**	**Joint sprain requiring medical intervention and healing**	**Injury requiring long-term intervention**
**Accidents or incidents**	**Dizziness during exercise requiring no medical intervention**	**Faintness requiring intervention**	**Cardiac distress requiring urgent intervention**
**Complaints**	**Subcutaneous infiltration of normal saline infusion**	**Subcutaneous infiltration of parenteral nutrient infusion fluids**	**Embolism from parenteral infusion**

We anticipate low incidence of adverse events as regular glucose is a naturally occurring and is metabolized in the same way as endogenous glucose. Subjects with overt cardiovascular or metabolic risk factors are excluded on the basis of preliminary health screens described in the previous section.

Reporting of SAEs, AEs, unexpected side effects, and ORIOs

We will follow the AE/ORIO reporting rules as defined by the University of Michigan IRB-MED (see the attached document AE/ORIO: Standard AE Reporting) that were modified to also meet NIDDK reporting requirements (see the attachment and table 3 below.)

Table 7: Reporting of AEs, SAEs or ORIOs to IRB-MED, GCRC, and NIDDK

Event type	Frequency of reports to SO	Frequency of reports to UM IRB-MED & GCRC), NIDDK
Serious or severe adverse events	Discussion with team physician within 24 hours	Report within 7 days of the event or notification of the event to UM
Moderate AE possibly, probably or related<br>Moderate AE unlikely or unrelated	Discussion with team physician within 72 hours	Report within 7 days of the event or notification of the event to UM<br>Report with annual report
Mild AEs regardless of relatedness	Notification at the time of monthly meeting	Report with annual report
ORIOs-urgent subject safety or regulatory concern	Discussion with team physician within 72 hours	Report within 7 days of the event or notification of the event to UM
ORIOs-situation/event potentially alters risk-benefit assessment or the integrity of the study	Discussion with team physician within 72 hours	Report within 7 days of the event or notification of the event to UM
ORIOs- all other	Discussion with team physician at the time of the monthly meeting	Report with annual report

(5) Stopping rules

Stopping rules will focus on the unlikely event of severe cardiac distress The study team, will evaluate and submit recommendation for stopping or continuing the study to the IRBMED and MCRU. The IRBMED will be the decision making committee. MCRU input will be submitted to the IRBMED.

G. REFERENCES CITED

Adam TC, Westerterp-Plantenga MS. Activity-induced GLP-1 release in lean and obese subjects. Physiol Behav 2004,83:459-66.

Berthoud HR. Vagal and hormonal gut-brain communication: from satiation to satisfaction. Neuroenterol Motil 2008, 20, Suppl 1: 64-72.

Borer KT, Wuorinen EC, Burant C. Exercise energy expenditure is not consciously detected due to oro-gastric, not metabolic, basis of hunger sensation. Appetite 2005, 45: 177-181.

Blundell JE, Levin F, King NA, Barkeling B, Gustafsson T, Hellstrom PM, Holst JJ, Naslund E. Overconsumption and obesity: peptides and susceptibility to weight gain. Regul Pept. 2008, 7;149: 32-8.

Borer KT, Wuorinen EC, Ku K, Burant C. Appetite responds to changes in meal content, whereas ghrelin, leptin, and insulin track changes in energy availability. J Clin Endocr Metab 2009,94: 2290-2298.

Cecil JE, Francis J, Read NW. Comparison of the effects of a high-fat and high-carbohydrate soup delivered orally and intragastrically on gastric emptying, appetite, and eating behaviour. Physiol Behav. 1999, 67:299-306.
Chanoine JP, Mackelvie KJ, Barr SI, Wong AC, Meneilly GS, Elahi DH. GLP-1 and appetite responses to a meal in lean and overweight adolescents following exercise.
Obesity 2008, 16: 202-4.
Edholm T, Degerblad M, Grybäck P, Hilsted L, Holst JJ, Jacobsson H, Efendic S, Schmidt PT, Hellström PM.Differential incretin effects of GIP and GLP-1 on gastric emptying, appetite, and insulin-glucose homeostasis. Neurogastroenterol Motil. 2010 [Epub ahead of print]
Flegal KM, Carroll MD, Ogden CL, Curtin LR. Prevalence and trends in obesity among US adults, 1999-2008. JAMA 2010, 303 (3): 235-241.
Heading RC, Nimmo J, Prescott LF, Tothill P. The dependence of paracetamol absorption on the rate of gastric emptying. Br J Pharmacol. 1973;47:415-21.
Johnson J, Vickers Z. Effects of flavor and macronutrient composition of food servings on liking, hunger and subsequent intake. Appetite.1993, 21:25-39.
King NA, Burley VJ, Blundell JE. Exercise-induced suppression of appetite: Effects on food intake and implications for energy balance. Eur J Clin Nutr 1994, 48: 715-724.
King NA, Caudwell PP, Hopkins M, Stubbs JR, Naslund E, Blundell JE. Dual-process action of exercise on appetite control: increase in orexigenic drive but improvement in meal-induced satiety. Am J Clin Nutr. 2009, 90:921-7.
Koeslag JH. Noakes TD, Sloan AW (1980) Post-exercise ketosis. J Physiol 301: 79-90.
Nauck MA, Niedereichholz U, Ettler R, Holst JJ, Orskov C, Ritzel R, Schmiegel WH. Glucagon-like peptide-1 inhibition of gastric emptying outweighs its insulinotropic effects in healthy humans. Am J Physiol Endocrinol Metab 1997;273:E981–8.
Saltin B, Wahren J, Pernow (1974) Phosphagen and carbohydrate metabolism during exercise in trained middle aged men. Scand J Clin Invest 33: 71-77.
